# Fast, Flexible, Feasible: A Transparent Framework for Evaluating eDNA Workflow Trade‐Offs in Resource‐Limited Settings

**DOI:** 10.1111/1755-0998.70091

**Published:** 2026-01-03

**Authors:** Yin Cheong Aden Ip, Elizabeth Andruszkiewicz Allan, Shana Lee Hirsch, Ryan P. Kelly

**Affiliations:** ^1^ School of Marine and Environmental Affairs University of Washington Seattle USA; ^2^ Human Centered Design and Engineering University of Washington Seattle USA

**Keywords:** basecalling algorithms, demultiplexing pipelines, DNA extraction, mesocosm, metabarcoding, nanopore sequencing

## Abstract

Environmental DNA (eDNA) analysis enables biodiversity monitoring by detecting organisms from trace genetic material, but high reagent costs, cold‐chain logistics and computational demands limit its broader use, particularly in resource‐limited settings. To address these challenges and improve accessibility, we directly compared multiple workflow components, including four DNA extraction methods, two primer sets, three Nanopore basecalling models, and two demultiplexing pipelines. Across 48 workflow combinations tested in an aquarium with 15 fish species, we mapped trade‐offs between cost, sensitivity, and processing speed to assess where time and resource savings are possible without compromising detection. Workflows using the Qiagen Blood and Tissue (BT) extraction kit and amplification using the MiFish‐U primer set provided the highest sensitivity, detecting ≥ 12 of 15 species by ~3–5 h and reaching the 15‐OTU plateau at ~8–10 h with Oxford Nanopore's high accuracy (HAC) basecalling model. Chelex, an alternative lower‐cost extraction method, showed partial recovery only (≤ 9 OTUs by 61 h) even with extended sequencing, and did not recover all 15 OTUs. DirectPCR and QuickExtract offered field‐friendly extraction alternatives that achieved comparable recovery in ~10–12 h, though their cost‐effectiveness varied. While the MarVer1 primer was designed to broaden vertebrate detection, it recovered the same fish species as MiFish‐U, though with fewer total reads. Real‐time sequencing trials (0–61 h) revealed that high‐efficiency workflows (BT + HAC) reached detection plateaus rapidly, indicating sequencing time can be reduced without sacrificing accuracy. The OBITools4 bioinformatics pipeline enabled automated demultiplexing but discarded more reads than an alternative, ONTbarcoder2.3, which retained low‐abundance taxa at the cost of manual curation. Rather than identifying a single ‘best’ workflow, this study provides a transparent decision framework for prioritising cost, speed, and sensitivity in eDNA applications, supporting scalable, cost‐effective eDNA monitoring in resource‐limited settings.

## 1 Introduction

Analysis of environmental DNA (eDNA) is revolutionising biodiversity monitoring by enabling species detection from trace genetic material in water, air and sediment, offering a powerful alternative to traditional survey methods (Deiner et al. 2017). While eDNA itself refers to the genetic material shed by organisms into the environment, its utility relies on robust and replicable analytical workflows. By capturing elusive or cryptic taxa that are often overlooked by conventional techniques (Ip et al. 2021), eDNA analysis has expanded the scope of ecological research and conservation efforts. However, its effectiveness depends on key methodological choices, including DNA extraction, primer selection, sequencing accuracy, and bioinformatics processing, which directly influence species detection rates and the comparability of results across studies (Andruszkiewicz Allan et al. 2025; Mathon et al. 2021; Tsuji et al. 2019).

Beyond these methodological considerations, eDNA work is constrained by logistical and technical challenges, particularly in remote field stations and under‐resourced laboratories. Many settings lack access to reasonably priced reagents, specialised laboratory equipment (e.g., benchtop sequencers, high‐speed centrifuges), cold‐chain storage, and reliable electricity, limiting the ability to process and analyse eDNA samples effectively (Kranzfelder et al. 2016; Majaneva et al. 2018; Hirsch et al. 2024). Additionally, eDNA metabarcoding workflows require extensive computational processing for analyses including basecalling, demultiplexing and taxonomic classification. This often exceeds the capacity of field‐based or small‐scale laboratories (Plewnia et al. 2025). For real‐time sequencing platforms, optimising sequencing duration is equally important, as continued sequencing may reach a plateau where additional runtime provides diminishing gains in species detection (Chang et al. 2024). Primer selection in resource‐limited settings presents a critical trade‐off: universal primers allow for broader taxonomic detection but may sacrifice species‐level resolution depending on the target gene region, while taxon‐specific primers can provide enhanced resolution within a group but limit the breadth of taxa detected. Consequently, researchers seeking both broad taxonomic diversity and species‐specific data must often sequence multiple marker regions, significantly increasing costs (Ficetola and Taberlet 2023). Thus, without clear guidance on marker selection, suboptimal primer choice can introduce taxonomic biases and reduce the reliability of biodiversity assessments (Shaffer et al. 2025).

To address these limitations, recent initiatives have focused on democratising eDNA methodologies, making them more accessible to eDNA‐users working with limited budgets and infrastructure. Portable sequencing platforms, such as Oxford Nanopore Technologies' (ONT) MinION, have been leveraged for on‐site sequencing and library preparation (Watsa et al. 2020; Chang, Ip, Ng, and Huang 2020; Kirchgeorg et al. 2024), and international standardisation efforts are starting to aim to optimise protocols for field‐deployable workflows without compromising data quality (Laamanen et al. 2025; Hirsch et al. 2024). Furthermore, a growing suite of user‐friendly extraction guidelines (Rieder et al. 2024) has lowered technical barriers and improved accessibility for new practitioners. To maximise the benefit of these advances, here we undertake a comparison of how methodological choices—from extraction and primer selection to basecalling and demultiplexing—affect species detection, cost‐efficiency and feasibility.

### 1.1 DNA Extraction

DNA extraction is one of the most resource‐intensive steps in eDNA workflows and influences every subsequent step in the analytical chain. The most commonly used eDNA extraction methods are commercial, column‐based kits, with ~50% of studies relying on Qiagen Blood & Tissue (BT) kits (Tsuji et al. 2019). These kits are favoured for their consistency and ease of use; however, their requirement for multiple buffers, wash steps and specialised equipment (e.g., centrifuge, vacuum manifold, incubator) often render them impractical for field applications. In contrast, simplified methods like Chelex‐based protocols offer an ultra‐low‐cost alternative (~$0.05 per sample; Holman et al. 2019) with minimal reliance on reagents and specialised equipment (heat block), though their susceptibility to PCR inhibitors can potentially reduce the detection of low‐abundance taxa in environmental samples (Walsh et al. 1991; Bracken et al. 2019; Karlsson et al. 2022). Other simplified extraction methods like QuickExtract (QE) and direct PCR (i.e., no extraction) represent intermediate solutions, requiring minimal infrastructure while maintaining moderate efficiency, but their performance can also vary in turbid or inhibitor‐rich samples (Majaneva et al. 2018; Lee‐Rodriguez et al. 2024; Scriver et al. 2024). Understanding how low‐cost, simplified extractions impact species recovery and where optimisations can mitigate losses is key to balancing feasibility of eDNA protocols with accuracy in species detection.

### 1.2 PCR Primers

Choosing the right primer set is fundamental to eDNA workflows, as it directly impacts detection sensitivity and the taxonomic resolution of generated data. For instance, MiFish‐U (Miya et al. 2015) is optimised for fish‐specific recovery, while broader‐coverage primers like MarVer1 (Valsecchi et al. 2020) target the same 12S region but were designed for wider vertebrate coverage (Doorenspleet et al. 2025; Plewnia et al. 2025; Shaffer et al. 2025). Notably, MarVer1 is known to amplify human DNA particularly well (Shaffer et al. 2025), potentially complicating analyses in remote or resource‐limited settings where strict contamination control is difficult. Different primer designs can influence the balance between taxonomic breadth and target specificity, an important consideration when choosing markers for multi‐taxon versus single‐group studies.

Beyond taxonomic specificity, primer choice is shaped by target amplicon length, which influences sequencing strategy and analytical feasibility. Both MiFish‐U and MarVer1 amplify fragments in the 150–200 bp range, a commonly targeted length in eDNA metabarcoding due to high PCR efficiency, broad applicability, and widespread use in short‐read metabarcoding workflows (Wang et al. 2023; Yang et al. 2024). In eDNA applications, shorter targets generally maximise detection sensitivity because shed DNA is fragmented (West and Deagle 2025). Recoverable copy number drops steeply as amplicon length increases (Brandão‐Dias et al. 2025). Although longer amplicons (> 650 bp) improve taxonomic resolution and phylogenetic inference, they introduce challenges related to amplification efficiency, library preparation, sequencing depth, and bioinformatics complexities (Chang et al. 2024; Deiner et al. 2017; Kirchgeorg et al. 2024; Tibone et al. 2025). Therefore, primer selection is a critical decision point that must balance specificity, taxonomic breadth, and sequencing constraints to optimise eDNA workflows for different ecological applications.

### 1.3 Downstream Processing

Post‐sequencing processes play a crucial role in eDNA analysis workflows as they influence the accuracy, efficiency, and scalability of taxonomic identification. Wet lab protocol choices, such as indexing strategies, adapter ligation and barcode designs, can affect demultiplexing efficiency, particularly in ONT workflows where basecalling accuracy affects barcode recognition (Krehenwinkel et al. 2019). Ensuring synergies between sequencing preparation and data processing strategies can help minimise barcode assignment errors and optimise downstream analyses (Petit‐Marty et al. 2023). Other post‐processing steps such as quality filtering, dereplication/denoising, and OTU/ASV clustering are standard in Illumina metabarcoding. For ONT amplicons, even when using the latest R10.4.1 chemistry, indel‐aware denoising/clustering remains comparatively computationally intensive. With earlier ONT chemistries, additional read‐polishing/consensus steps were often needed (Chang, Ip, Bauman, and Huang 2020), which are generally impractical in field settings. Since our goal is a real‐time, resource‐limited pipeline, we adopted a minimal path: demultiplexing followed by direct k‐mer–based classification (Kraken2) rather than an intermediate clustering step (Lepuschitz et al. 2020; Lu et al. 2022; Bayer et al. 2024). While long‐read, indel‐aware clustering programmes (e.g., amplicon_sorter; Vierstraete and Braeckman 2022) are promising, they typically demand server‐class hardware and longer runtimes, and were therefore outside the scope of the resource‐limited workflows in this study.

Unlike Illumina sequencing, which outputs final nucleotide sequences (AGCT) directly, ONT instruments measure a time‐series of ionic current through each pore. These current traces are then converted to nucleotide sequences by neural network basecallers (Wick et al. 2019). ONT offers three basecalling models—Fast, High Accuracy (HAC), and Super Accuracy (SUP), which allow users the option to balance computational efficiency and read fidelity (Oxford Nanopore Technologies 2025). These differences directly impact taxonomic resolution and the likelihood of achieving species‐level identification (Wick et al. 2019; Chang et al. 2024). Demultiplexing influences detection outcomes by determining the efficiency with which reads are assigned to their respective samples. Tools such as ONTbarcoder2.3 and OBITools4 handle read errors, indels, and barcode mismatches differently, resulting in trade‐offs between automation, read retention, and the need for manual intervention (Chang et al. 2024). While permissive tools may preserve a higher number of reads, they often introduce greater workflow overhead, requiring manual intervention and multiple systems to process many files concurrently, whereas more automated pipelines reduce such labour even if they discard a small proportion of reads. The optimal demultiplexing strategy thus depends on the dataset complexity, available resources, and the desired level of accuracy.

Following demultiplexing, taxonomic classification also influences workflow efficiency, as differences in classification algorithms, reference database completeness, and error tolerance can impact species identification and detection sensitivity. Curated reference databases like MitoFish (Zhu et al. 2023) enhance classification accuracy by providing curated and taxonomically comprehensive resources for species assignment. To support rapid, high‐throughput analysis of large ONT datasets, we selected Kraken2 as our taxonomic classifier because of its computational efficiency and compatibility with custom reference databases (Zhu et al. 2023; Bayer et al. 2024; Liu et al. 2024; Lepuschitz et al. 2020; Lu et al. 2022). These bioinformatic decisions are integral to balancing performance and scalability in eDNA metabarcoding workflows, aligning metabarcoding outcomes with the constraints of available computational resources and research priorities.

**FIGURE 1:**
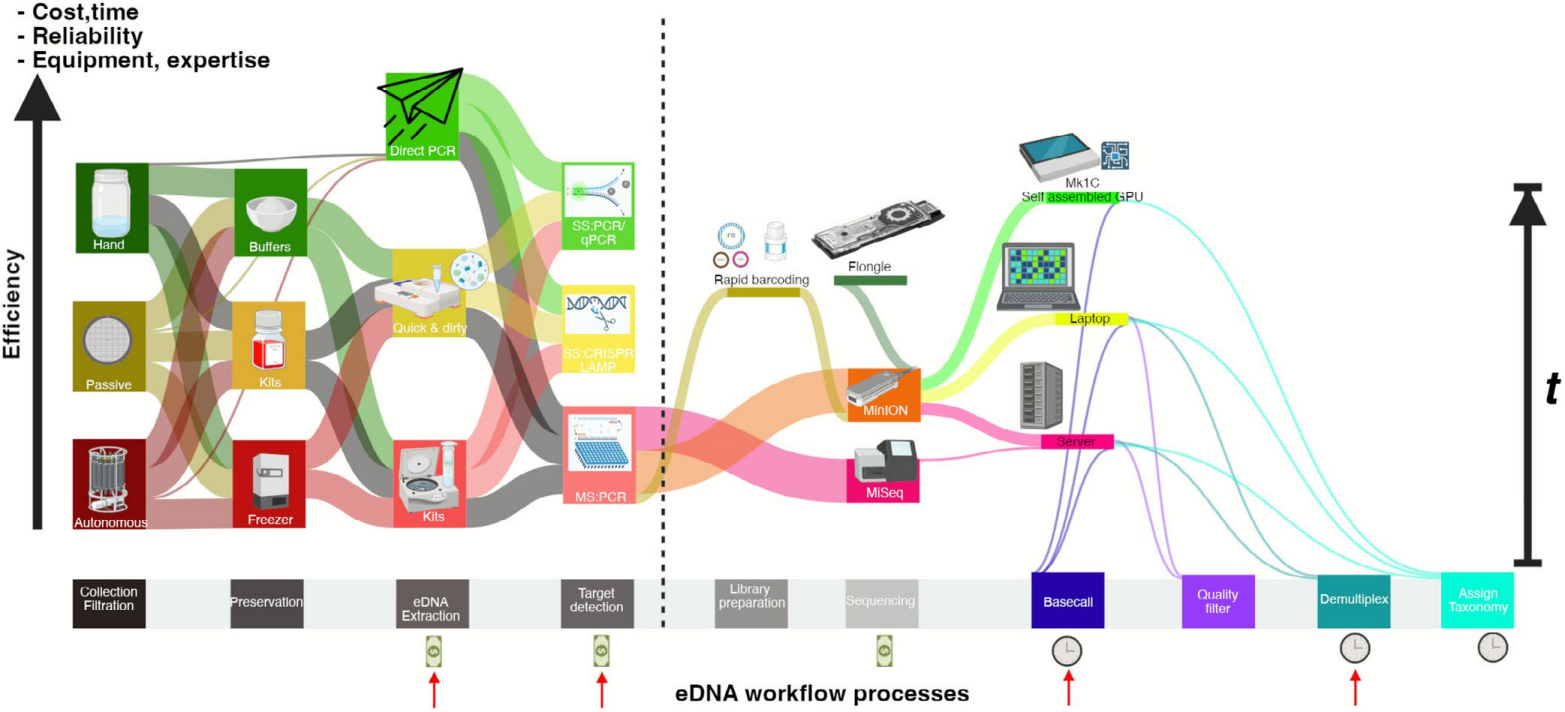
Conceptual overview of eDNA workflows and trade‐off considerations. This figure illustrates the eDNA workflow from sample collection to taxonomic assignment, highlighting key trade‐offs in cost, time, reliability and equipment requirements. The vertical axis represents a composite efficiency metric (e.g., cost, time and resources per taxon detected), with streamlined, field‐friendly methods at the top and resource‐intensive workflows at the bottom, while the horizontal axis tracks sequential eDNA processing steps. Red arrows indicate the methodological steps tested in this study, that is, eDNA extraction, target detection (broad vs. specific primers), basecalling models and demultiplexing workflows; each accompanied by red arrows with cash or clock symbols to denote opportunities for cost savings or time optimisation. Sequencing (MinION Mk1B; R10.4.1) and taxonomic assignment (Kraken2) were selected for their affordability and speed but were not directly tested in this study, and thus, do not feature red arrows. These workflow optimisations, including simplified extraction, Nanopore sequencing as a cost‐effective alternative to the more common Illumina MiSeq and accelerated bioinformatics pipelines for taxonomic classification, enhance the feasibility of eDNA analysis, particularly in resource‐limited settings. Elements not analysed empirically in this study are shown as illustrative options (e.g., MinION Mk1C) and were not benchmarked here.

This study directly compares the trade‐offs in eDNA workflows, identifying how methodological ‘shortcuts’ for cost‐effectiveness and time‐efficiency remain viable and where they introduce meaningful losses in resulting data quantity and quality (Figure 1). We compare four DNA extraction methods (Qiagen BT, Chelex, QuickExtract and DirectPCR) with two 12S primer sets (MiFish‐U and MarVer1), three Nanopore basecalling models (FAS, HAC and SUP), and two demultiplexing pipelines (OBITools4 and ONTbarcoder2.3), resulting in 48 unique workflow combinations tested in a controlled aquarium containing 15 known fish species. In addition to endpoint analyses, we examined species accumulation trends in real‐time sequencing by analysing ONT data at hourly intervals up to 61 h, assessing how sequencing duration can be reduced without sacrificing accuracy. By comprehensively assessing detection accuracy and computational efficiency, we provide a decision framework for researchers to tailor eDNA workflows based on specific priorities, whether they be for maximising species recovery, minimising costs, or enabling rapid field‐based analyses. Ultimately, these findings contribute to democratising eDNA‐based biodiversity monitoring, ensuring that cost‐effective and scalable approaches remain accessible for conservation, invasive species management, and ecological research, particularly in resource‐limited settings.

## 2 Materials and Methods

### 2.1 Experimental Design and Selection of eDNA Extraction Methods

We employed a factorial design to assess the effects of various methodological choices on eDNA metabarcoding outcomes. In total, we processed 12 biological samples (three replicates per extraction across four methods) using two primer sets, three basecalling models, and two demultiplexing pipelines, resulting in 48 unique workflow combinations. Applied across the 12 biological replicates, this produced 144 dataset instances for analysis. This design enabled the evaluation of both individual factor effects and potential interactions. To account for variability, we included biological replicates (three replicate water samples per extraction method) and technical replicates (three PCR replicates per sample, aggregated at the PCR level). This replication strategy facilitated a robust assessment of workflow consistency.

Four DNA extraction methods were evaluated for feasibility and efficiency in resource‐limited contexts (Table 1):

1. DNeasy Blood & Tissue (BT) (Qiagen, Hilden, Germany): A widely used column‐based approach (Tsuji et al. 2019) that provides high DNA recovery and purity but is expensive, requires multiple reagents and specialised laboratory equipment, such as a high‐speed centrifuge or a vacuum manifold with an external pump, and an incubator at 56°C for Proteinase‐K lysis.
2. Chelex‐100 (Chelex) (Bio‐Rad, Hercules, CA, USA): A low‐cost resin‐based method that can reduce per‐sample costs to approximately $0.05 (Bracken et al. 2019) and requires minimal equipment (e.g., portable heat block) but sometimes exhibits reduced sensitivity to rare taxa due to inhibitor presence (Karlsson et al. 2022).
3. QuickExtract (LGC Biosearch Technologies, Middleton, WI, USA): An enzymatic lysis approach requiring minimal equipment (e.g., portable heat block) and moderate cold storage. It has proven effective for small insect exuviae (Kranzfelder et al. 2016) and greenhouse arthropod eDNA (Lee‐Rodriguez et al. 2024) but occasionally retains inhibitors from turbid samples (Majaneva et al. 2018).
4. DirectPCR/Centrifugal Dialysis (MilliporeSigma, Burlington, MA, USA): Relies on concentrating resuspended water into smaller volumes and enabling direct amplification of DNA from environmental samples without purification. It does not require reagents or cold storage and is promising for field applications, though performance can vary in low‐DNA contexts, and it does need a mini‐centrifuge (Ip et al. 2024; Kirtane and Deiner 2024).

**TABLE 1:** Comparison of cost, processing time, and equipment requirements for each extraction method.

Extraction Method	~Cost (USD)	Time	Reagent Requirements	Cold Storage	Specialised Equipment	Yield	Purity
BT	$4.54	4	High	No	High‐speed centrifuge/vacuum manifold, incubator	High	High
Chelex	$0.10	3	Low	No	Heat block	Low	Low
QuickExtract	$4.70	2	Low	Moderate	Heat block	Medium	Low
DirectPCR	$6.00	1	None	None	Mini‐centrifuge	Medium	Low

*Note:* ‘Time’ is expressed in relative units representing processing speed. ‘Cold storage’ refers to storage of extraction reagents required by each kit. Costs are USD estimates (2024–2025) from current institutional quotes. BT cost excludes molecular‐grade ethanol, which is not supplied with the kit and increases the true per‐sample cost. DirectPCR cost is dominated by the Amicon Ultra‐0.5 50 kDa filter. Prices vary by supplier/region.

### 2.2 Sample Collection, Filtration and Preservation

To provide a controlled setting for evaluating different eDNA methods and species detection rates, water samples were collected from the Seattle Aquarium (Seattle, WA, USA). The facility houses a 450,000 L mixed‐species aquarium containing 19 known fish species (see Table S1), which provides a standardised setting for workflow comparisons. For downstream analyses we report detections at the Operational Taxonomic Unit level (15 OTUs) because two species pairs are indistinguishable with the 12S markers used here and are therefore collapsed. The full species list and species–OTU mapping are provided in Table S1. All detections are reported at OTU resolution unless stated otherwise.

A 20 L water sample was taken from the surface of the aquarium tank using a 30 L carboy pre‐treated with 10% bleach, rinsed with DI water, and handled with gloves to minimise contamination. Filtration was performed on site using a Smith‐Root Citizen Science Sampler and filtration housings equipped with 5.0 μm mixed cellulose ester (MCE) filters (Allan et al. 2023). Additionally, five field‐negative filtration blanks (1 L of Milli‐Q water each) were processed using the same system. In total, 15 L of aquarium water was filtered (1 L per replicate) within 30 min to ensure all samples were filtered and processed under similar conditions, minimising potential biases from DNA degradation. To maintain homogeneity, the 30 L carboy was thoroughly shaken before each transfer. Filters were then stored on ice for transport to the laboratory for immediate DNA extraction.

### 2.3 Sample Processing, eDNA Extraction and Direct PCR Preparation

Before DNA extraction, filters were carefully transferred with sterile, bleach‐treated forceps into 5 mL tubes containing 1.5 mL of Milli‐Q water. The filters were randomised across DNA extraction treatments to include three biological replicates of aquarium water and one field blank per extraction method. Each filter was vortexed for 1 min to resuspend eDNA. For DirectPCR, 500 μL of the resuspended water was transferred to a 50 kDa molecular‐weight‐cutoff Amicon spin column and centrifuged at 14,000 g for 1 min each, repeated three times to process the total volume of 1.5 mL. The resulting concentrate (50–100 μL) was eluted by inverting the spin column and centrifuging it at 1000 g for 3 min, then used directly for PCR. Note that both DirectPCR and BT require a centrifuge. However, DirectPCR is a single‐step spin with no kit buffer handling, so hands‐on time is minimal, and its per‐sample consumable cost is largely the Amicon filter (overall cost comparable to BT once ethanol is included). In contrast, for BT, Chelex and QuickExtract, manufacturer protocols were followed with minor eDNA‐specific optimisations (see Supporting Information S1). Extractions were performed in two sequential batches: BT and DirectPCR were processed simultaneously, followed by QuickExtract and Chelex. All extractions were completed within 2–4 h after sample collection. Extracts were stored at −20°C until PCR amplification and sequencing, which occurred within three months.

### 2.4 Library Preparation and Sequencing

Amplicons were generated using two primer sets, MiFish‐U (a 12S primer targeting fish; Miya et al. 2015) and MarVer1 (a broader 12S primer for vertebrates and metazoans; Valsecchi et al. 2020). We compared MiFish‐U (fish‐focused) with MarVer1 (broader vertebrate scope) primers to demonstrate typical use‐cases, such as dedicated fish surveys versus broader vertebrate assessments. Even in closed systems, trace non‐fish vertebrate DNA (e.g., as a result of fish handling or feed) can occur, so this comparison asks whether broader coverage dilutes fish‐specific depth without improving fish detections. As our study focuses on fish community detection using field‐portable analysis, downstream classification was restricted to Actinopterygii. The primer sequences were as follows: MiFish‐U Forward: 5′‐GCCGGTAAAACTCGTGCCAGC‐3′; MiFish‐U Reverse: 5′‐CATAGTGGGGTATCTAATCCCAGTTTG‐3′; MarVer1 Forward: 5′‐CGTGCCAGCCACCGCG‐3′; MarVer1 Reverse: 5′‐GGGTATCTAATCCYAGTTTG‐3′. To differentiate samples and reduce multiplexing errors, we attached unique 5′ tags (also known as indices or barcodes) to each primer. Tags were designed based on unpublished guidance provided by Eric Coissac (personal communication). In accordance with confidentiality requirements, we do not provide sequence‐level details, design constraints, or the tag‐design code.

Each PCR reaction was performed in triplicate with a total volume of 20 μL, consisting of 10 μL 2× Phusion Mix (Thermo Fisher Scientific), 0.6 μL DMSO (3% final concentration), 0.5 μL BSA (0.5 μg/μL final concentration), 3.9 μL molecular‐grade water, 0.5 μL each of forward and reverse primers (10 μM), and 5 μL of eDNA extract/DirectPCR concentrate. PCR cycling conditions followed Shaffer et al. (2025). Each PCR replicate used a unique forward–reverse 14‐bp tag pair. Tagged replicates from the same biological sample were pooled prior to library preparation, and replicate identity was recovered later by tag‐based demultiplexing. Amplicons were pooled without equimolar normalisation and purified using AMPure XP beads (Beckman Coulter) at a 0.7× ratio to remove smaller non‐target fragments. This non‐normalised pooling approach was intentional, designed to evaluate integrated workflow performance under field‐realistic constraints where higher‐yield extracts naturally contribute more molecules to sequencing, reflecting the practical throughput differences researchers experience when choosing extraction methods in resource‐limited settings. In principle and all else being equal, workflows with cleaner extractions and more efficient amplification reactions would yield greater concentrations of amplicons, although testing these details remains beyond the scope of the present manuscript.

Library preparation was carried out following the SQK‐LSK‐114 ligation sequencing kit protocol (Oxford Nanopore Technologies). The completed library was loaded onto an R10.4.1 MinION flow cell and sequenced on a MinION Mk1B platform using MinKNOW v24.06.16 (Windows 11), with the run lasting up to 61 h. The MinION Mk1C was not evaluated because our benchmarking targets assay and analysis components that are independent of instrument packaging.

### 2.5 Basecalling Models

Raw POD5 reads were basecalled using Dorado v0.9.1 to evaluate three Nanopore basecalling modes: Fast, High‐Accuracy (HAC) and Super Accurate (SUP). Basecalling was performed on two hardware setups: (i) An Apple MacBook Pro 2023 (M3 Max; 16 CPU cores [12 performance + 4 efficiency]; 40‐core integrated GPU; 64 GB unified memory), to assess accessibility and processing times on a more consumer‐friendly, widely available system; (ii) An MSI Raider 18 HX Gaming Laptop (Windows 11 Intel Core i9‐14900HX (32 logical cores), NVIDIA GeForce RTX 4090 Laptop GPU (16 GB VRAM), and 16 GB RAM available to the WSL2 environment), which was used for all final analyses due to its higher computational power. Each dataset was basecalled using all three models for comparison. This comparison provides insight into the performance differences between Apple silicon processors and high‐end NVIDIA GPUs, helping users weigh trade‐offs between hardware accessibility and computational efficiency. For downstream comparisons, demultiplexers were evaluated on the identical basecalled FASTQ inputs produced by each model (Fast/HAC/SUP) to isolate demultiplexing effects from basecalling.

### 2.6 Demultiplexing Pipelines

Demultiplexing was performed using two distinct tools designed for ONT sequencing: ONTBarcoder2.3 (Srivathsan et al. 2024), a commonly used programme to process amplicon barcoding libraries on Nanopore platforms, and OBITools4 (https://github.com/metabarcoding/obitools4), which was tested here as an alternative indel‐aware tool. Reads were demultiplexed per tag, yielding separate FASTQ files per PCR replicate. ONTBarcoder2.3 is widely adopted for its ability to handle barcode‐based demultiplexing of Nanopore amplicon data, including detection of self‐ligated reads which occur when multiple amplicons become joined during sequencing but are initially processed as a single read. These reads are recognised and split, allowing more usable sequences to be retained for downstream analysis (Chang et al. 2024). In our workflow, ONTBarcoder2.3 was run with a minimum read length of 200 bp and an expected amplicon length of 250 bp, while other parameters were kept at default (Supporting Information S2). Here, ‘expected amplicon length’ refers to the full read prior to demultiplexer trimming (insert + primers + tags). These settings filter truncated reads while retaining full‐length amplicons for both primer sets. Next, the standard genetic code was selected, and only sequences deviating by up to 2 bp from the tag sequence were retained. This threshold was supported by the tag design, which ensured a minimum difference of ≥ 3 bp between barcode tags to reduce read misassignments. However, ONTBarcoder2.3 is graphical user interface (GUI) based and does not support command line execution, limiting its utility in high‐throughput workflows.

OBITools4 was evaluated in this study for its ability to handle insertions and deletions (indels) inherent to Nanopore sequencing data. For OBITools4, we used the *obimultiplex* module, specifying an indel‐aware demultiplexing process with a maximum mismatch threshold of two base pairs (Supporting Information S3). The output from OBITools4 is a single concatenated FASTQ file, with sample information embedded in each sequence header. To organise the data at the sample level, we implemented a custom Python script to parse the sequence headers and split the reads into separate FASTQ files corresponding to individual samples.

To ensure accurate separation of reads originating from each primer set, demultiplexing was performed twice, once for MiFish‐U and once for MarVer1. Both programmes produced outputs that had been trimmed of primers and tags as part of the demultiplexing process, ensuring that only high confidence reads progressed downstream for taxonomic classification.

For comparability, both demultiplexers were run on the same basecalled FASTQ inputs generated by each Dorado model (Fast/HAC/SUP). Complete command lines and parameter files are provided in Supporting Information S3 and S4. This design attributes observed differences to demultiplexing rather than basecalling. Note that Dorado (v0.9.1) also offers integrated demultiplexing via user‐defined barcode .*toml* files, but we did not evaluate that feature here. To maintain a modular, version‐pinned, and offline‐reproducible comparison, we benchmarked ONTbarcoder2.3 and OBITools4. As configuration and behaviour stabilise, Dorado demultiplexing is a promising option for streamlining future work.

### 2.7 Taxonomic Classification

We did not perform clustering or read polishing because consensus‐calling or indel‐aware clustering for ONT amplicons remains comparatively computation‐intensive. With earlier ONT chemistries, it often benefits from additional consensus steps, making it poorly suited to resource‐limited or field contexts. Assuming the user has an existing database, a k‐mer–based classifier provides rapid, lightweight assignment without an intermediate clustering stage (Lepuschitz et al. 2020; Lu et al. 2022; Bayer et al. 2024).

To assign species annotations, all sequence classification was conducted with Kraken2 v2.1.2 using a custom MitoFish database (Wood et al. 2019; Zhu et al. 2023). Kraken2 employs a k‐mer–based approach that is computationally more efficient than BLAST (Lepuschitz et al. 2020; Lu et al. 2022), thus making it more practical for field deployment and for users with limited computational resources (Plewnia et al. 2025). Recent simulation studies further suggest that Kraken2, when paired with a comprehensive or curated database and a modest confidence threshold (0.05–0.2), achieves a balance between sensitivity and specificity (Liu et al. 2024; Bayer et al. 2024).

For this study, a custom Kraken2 database was constructed by downloading curated mitochondrial sequences for fish species from the MitoFish repository (http://mitofish.aori.u‐tokyo.ac.jp). Each sequence was assigned a valid NCBI taxonomic identifier and integrated with the NCBI taxonomy database using the *kraken2‐build* function. The database was validated using *kraken2‐inspect* and cross‐verified against BLAST‐based classifications to ensure accuracy. Of the 19 named aquarium species, 16 have 12S records in MitoFish. *Rhinogobiops nicholsii* , *Hemilepidotus spinosus* and *Sebastes diaconus* lack 12S entries. Because the latter two are congeners of *H. hemilepidotus* and *S. flavidus* with near‐identical 12S, we analysed detections at the OTU level by collapsing those pairs (see Table S1).

We applied a confidence score threshold of 0.1 and retained reads mapped to Actinopterygii in both the MiFish‐U and MarVer1 datasets for downstream analysis. To mirror typical field practice and maintain generality, we did not generate aquarium‐specific reference sequences; all assignments used the public MitoFish database (downloaded 8 November 2024). The known species list was used only to evaluate detections and to collapse indistinguishable 12S pairs into operational taxonomic units (OTUs) when multiple named taxa share effectively identical 12S sequences for both primer sets. Ambiguous assignments were retained at genus level. Here, we define an OTU as a taxonomy‐defined unit: a species when resolvable by 12S; otherwise, they are a collapsed species pair or genus. Reads were not clustered or consensus‐called; counts were summed by their final Kraken2 taxon label. A two‐column mapping from raw Kraken2 calls to curated OTUs is provided. Raw read compositions and relative abundances from the Kraken2 results were used for subsequent statistical analyses. Potential genus‐level ambiguities, such as those observed in *Sebastes* spp., were manually flagged for further verification (Table S1). Accordingly, we report detections at OTU resolution; the two missing congener references did not change the evaluable target count, and the absence of *R. nicholsii* from public databases prevented in silico primer checks for that species.

### 2.8 Real‐Time Sequencing

In addition to the consolidated POD5 files mentioned in Section 2.5, the hourly POD5 data from the 61‐h run were processed separately to enable a time‐resolved analysis of eDNA detection. ‘Hour’ denotes cumulative sequencing time on the flow cell; basecalling, demultiplexing, and taxonomic assignment were performed offline on per‐hour POD5 bins. After each hourly interval, the corresponding POD5 file was transferred to the MSI Raider 18 HX laptop for basecalling (Fast, HAC, SUP), followed by OBITools4 demultiplexing (leveraging its command‐line automation) and Kraken2 classification as described in Section 2.7. This produced 61 hourly snapshots of eDNA readouts, enabling the construction of species accumulation curves that identified the minimum run time needed to recover all known aquarium species. Additionally, species detection frequencies were tracked hourly to assess how abundance and sequencing duration influenced detection frequencies. We exported the MinKNOW run report for the 61‐h sequencing run and summarised cumulative reads and bases over time.

### 2.9 Statistical Analysis and Visualisation

All statistical analyses were conducted in R Studio (Version 2024.04.1 + 748, R version 4.2.0) to evaluate how extraction method, primer choice, basecalling model, demultiplexing strategy, and sequencing duration influenced eDNA‐based species detection. Raw DNA read counts for the known 15 aquarium Operational Taxonomic Units (OTUs; species‐equivalent units), obtained from Kraken2 outputs, were log_10_‐transformed to reduce skewness and used for bar plots or summary metrics. All inference was performed in proportion (frequency) space using the zero‐ and one‐inflated Dirichlet (ZOID) regression model (Jensen et al. 2022), which allowed us to carry out a straightforward regression analysis on compositional data. Raw counts were log_10_‐transformed only for visualisation purposes; statistical models used proportions exclusively.

Specifically, we employed a partial‐aggregation approach in which PCR replicates (three per biological replicate) were collapsed after demultiplexing by summing species read counts across PCRs, yielding one count vector per biological replicate; these were then converted to proportions for modelling. Biological replicates (bio_rep) were retained as distinct independent observations to estimate factor‐level effects on species detection probabilities. The figures therefore display one datapoint per biological replicate (PCR‐collapsed). Within each biological replicate (with its three PCR replicates averaged), proportions were derived by dividing each OTU's read count by the total reads of all 15 target OTUs, ensuring the sum of proportions equaled one. In the ZOID design matrix, Qiagen BT extraction, MiFish‐U primers, HAC basecalling and ONTbarcoder2.3 demultiplexing were set as the baseline reference levels. In addition, we compared this partial‐aggregation with a fully aggregated approach, in which all replicates (biological and PCR) are averaged post‐normalisation. This comparison allowed us to assess the impact of aggregation strategy on model outputs; the fully aggregated analysis is presented in Supporting Information S4, while the partial‐aggregation approach is used as our primary analysis because it better preserves inherent biological variability. We generated bivariate, forest, and box plots to visualise factor‐level effects across species detections, extracted posterior coefficients for each species‐factor combination, and performed posterior predictive checks to assess model fit and zero‐inflation mismatches (Figure S6). To capture potential nonlinear trends in species accumulation over sequencing time, we fitted Generalised Additive Models using the mgcv package (Wood 2001) with sequencing time as a thin‐plate regression spline smooth term.

Hierarchical Bayesian inference was carried out with the zoid package (Jensen et al. 2022), which runs Stan under the hood via rstan (McElreath 2018). Nonlinear species‐accumulation trends were fitted with GAMs using mgcv (Wood 2001). Data wrangling and visualisation relied on the tidyverse suite (Wickham et al. 2019), including dplyr, tidyr, purrr and stringr. We used ggplot2 and the ggsci extension for plotting, and ggpubr to add statistical annotations.

## 3 Results

Across all workflows, 17 of the 19 expected fish species were detected. Two species, *Rhinogobiops nicholsii* and *Sebastes nebulosus* , were not detected in any workflow, likely due to low eDNA concentrations. BLAST analyses indicate that both primers align well with the 12S region in *Sebastes nebulosus* , which is present in the MitoFish database, suggesting that primer mismatches are unlikely to explain its non‐detection. In contrast, *Rhinogobiops nicholsii* is absent from both MitoFish and GenBank databases, precluding in silico verification of primer compatibility; its non‐detection is likely attributed to low template levels. Additionally, two species pairs were grouped due to near‐identical 12S sequences for both primer sets, limiting taxonomic resolution: (i) *Hemilepidotus hemilepidotus* with *Hemilepidotus spinosus* and (ii) *Sebastes flavidus* with *Sebastes diaconus*. Notably, both *Hemilepidotus spinosus* and *Sebastes diaconus* are also missing from the MitoFish database, which may have contributed to the merging of these taxa. This resulted in a final curated dataset of 15 Operational Taxonomic Units (OTUs; species‐equivalent units) (see Table S1). These manual merges reflect genuine marker‐level indistinguishability in public references and represent a standard, transparent curation step in eDNA metabarcoding, not a system‐specific correction. We consider this count as our maximum number of OTUs reasonably detectable under the constraints of the primer choice, reference database completeness, and the resolution limits of the 12S marker.

### 3.1 Read Recovery and Species Detection

#### 3.1.1 Effect of Extraction Method on Read Depth and Species Detection

As noted above, we used unnormalised read‐counts as our core outcome metric because this best captures the cumulative efficiency of the various steps in the workflow; we are interested in the effects of different workflow decisions on outcome datasets, rather than the ways in which those outcome datasets do or do not reflect unbiased estimates of underlying biomass or other ecological parameters (about which see, e.g., Shelton et al. 2023). This is in contrast to the standard Illumina bioinformatics processes, in which normalised read counts are best understood in proportion space (see Discussion, below).

**FIGURE 2:**
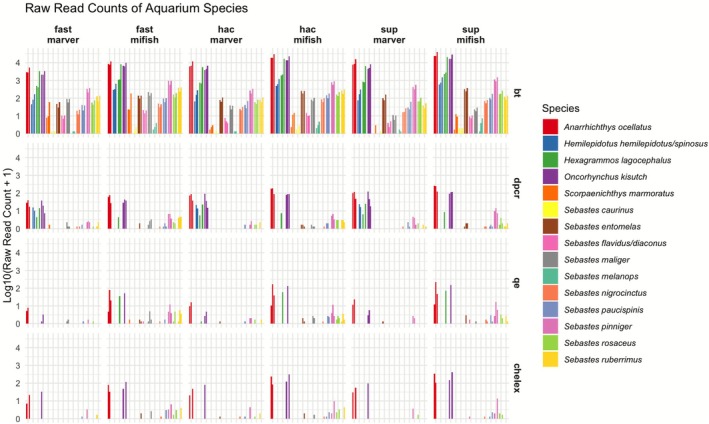
Read Counts Across Workflow Variables in eDNA Analysis. Bar plots display the log_10_‐transformed raw read counts of aquarium species detected across various workflow components, including basecalling models (Fast, HAC and SUP), primer sets (MiFish‐U and MarVer1) and DNA extraction methods (BT, Chelex, QuickExtract and DirectPCR). Only results from the ONTbarcoder2.3 demultiplexing approach are shown here; full results comparing OBITools4 are provided in the Supporting Information. Biological replicates (bt1, bt2, bt3) are shown separately. Within each biological replicate, PCR replicates (a/b/c) were uniquely tagged, pooled for sequencing, demultiplexed, and species read counts were summed across PCRs before plotting. Each species appears as up to three side‐by‐side bars, or fewer if not detected in all replicates.

Among the extraction methods, Qiagen BT consistently yielded the highest read counts across all conditions (Figure 2), with BT‐extracted samples producing ~420,000 raw reads per sample replicate in the best workflows. The highest recovery was observed under the SUP basecalling + MiFish‐U primers + ONTbarcoder2.3 workflow. In contrast, Chelex consistently exhibited the lowest read counts, with most samples receiving fewer than ~1000 reads, reducing the ability to recover low‐abundance taxa across all workflows. The best‐performing Chelex workflow (HAC + MiFish‐U + ONTbarcoder2.3) recovered only ~3000 reads per run, nearly an order of magnitude lower than the lowest‐throughput Qiagen BT workflows. DirectPCR and QuickExtract yielded intermediate read depths, typically ~10,000–100,000 reads, offering moderate yields that occasionally approached BT levels when paired with optimal primer and basecalling choices.

**FIGURE 3:**
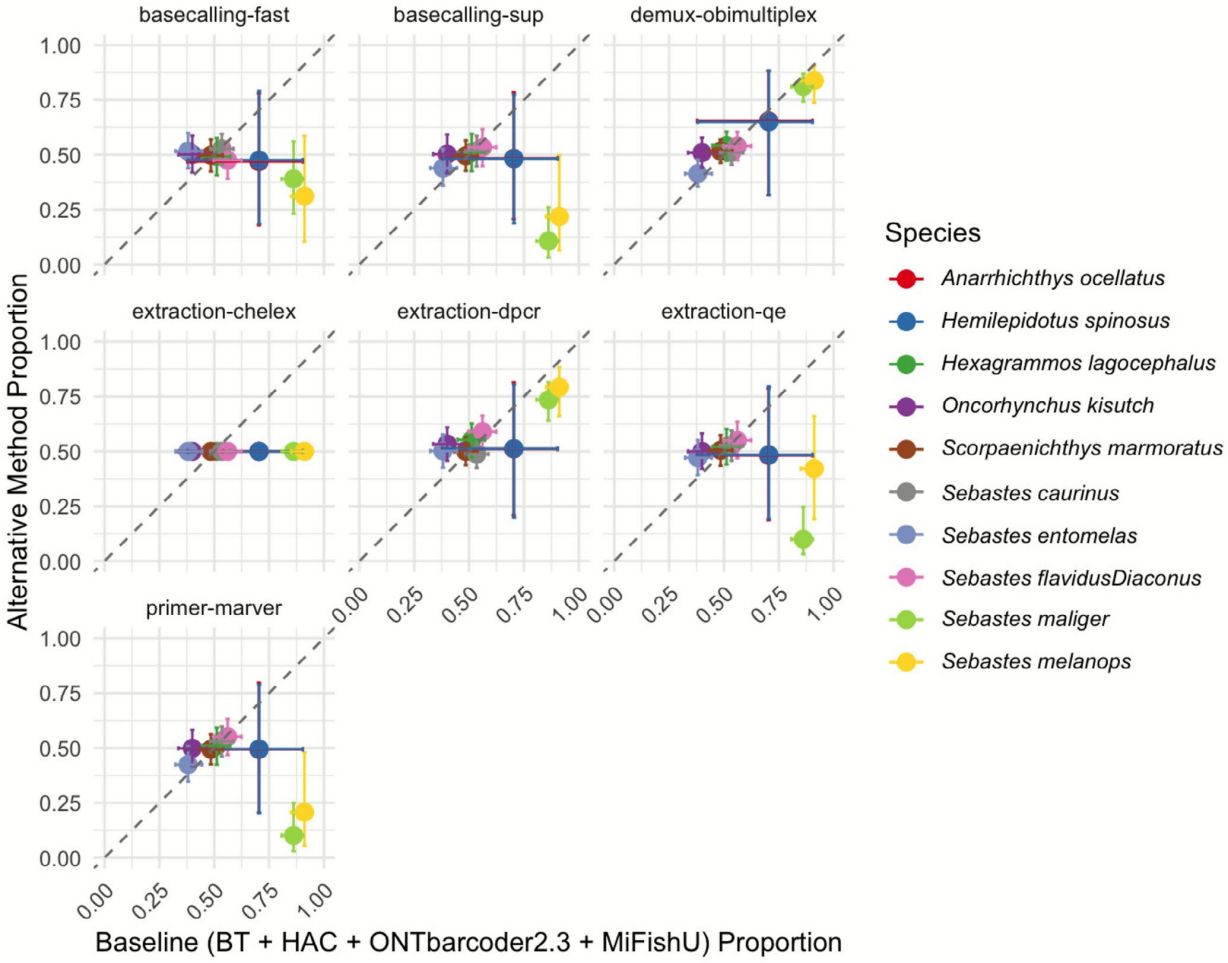
Bivariate Comparison of Predicted Species Detection Proportions. Each panel shows the predicted detection proportion for an alternative method (y‐axis) compared to the baseline configuration (BT + HAC + ONTbarcoder 2.3 + MiFishU; x‐axis). Points represent the posterior mean predicted proportion for each species, and error bars represent the 95% credible intervals. The dashed diagonal line (y = x) indicates equal detection between the baseline and alternative methods. Points above the diagonal denote higher detection probability under the alternative method, while points below indicate lower detection probability.

These patterns are further illustrated in the bivariate comparison of predicted detection proportions from ZOID coefficients (Figure 3, Table 2), where extraction methods show distinct relationships to the baseline BT workflow. Chelex samples consistently fall below the diagonal identity line, demonstrating uniformly reduced detection across all species—a pattern suggesting systematic DNA loss or inhibitor carryover that affects all taxa similarly regardless of abundance. In contrast, DirectPCR samples cluster tightly around the diagonal, indicating performance comparable to BT for most species with only minor variations. QuickExtract shows an intermediate pattern, indicating reduced detection efficiency for certain taxa relative to BT (e.g., *Sebastes maliger* ). Notably, Chelex results change when biological replicate variability is preserved, highlighting that it is highly variable and unreliable compared to BT. DirectPCR and QuickExtract showed neutral to mildly positive coefficients across several species, underscoring their potential as practical, cost‐effective alternatives when maximum sensitivity is not critical.

**TABLE 2:** Example output of Zero‐and‐One Inflated Dirichlet (ZOID) posterior estimates for a single species ( *Anarrhichthys ocellatus* ), showing the posterior mean and 95% credible intervals for methodological factors relative to the baseline workflow (Qiagen BT, MiFish‐U, HAC, and ONTbarcoder 2.3).

Species	Factor	Posterior mean	95% Credible interval (lower, upper)
*Anarrhichthys ocellatus*	(Intercept)	0.8637	(−0.5009, 2.2376)
	bio_rep2	−0.0891	(−1.5387, 1.3084)
	bio_rep3	0.2489	(−1.1689, 1.7096)
	demux‐obimultiplex	0.6384	(−0.7768, 2.0065)
	primer‐marver	−0.0241	(−1.3516, 1.3701)
	basecalling‐fast	−0.1230	(−1.5233, 1.2630)
	basecalling‐sup	−0.0648	(−1.3421, 1.2925)
	extraction‐dpcr	0.0453	(−1.3289, 1.4842)
	extraction‐qe	−0.0731	(−1.4720, 1.3047)
	extraction‐chelex	0	(0, 0)

*Note:* Full results for all 15 species and all factor combinations are provided in Supporting Information S4.

To verify that conclusions were not dependent on unequal amplicon pooling, we performed two additional sensitivity analyses: (i) rarefaction to equal read depth and (ii) presence–absence transformation (Figure S9). Both approaches yielded the same extraction method ranking (BT>DirectPCR~QuickExtract>Chelex), with differences remaining statistically significant (Kruskal–Wallis *p* < 0.001).

#### 3.1.2 Effect of Primer, Basecalling Algorithm and Demultiplexing on Read Depth and Species Detection

Primer selection strongly influenced read recovery, with MiFish‐U consistently outperforming MarVer1 across workflows (Figure 2). MiFish‐U samples generated significantly higher raw read counts, reaching ~50,000 to 300,000 reads per sample replicate compared to ~3000–20,000 from MarVer1 (Figure 3). Notably, *Sebastes rosaceus* and *Sebastes ruberriumus* showed particularly reduced detection with MarVer1 primers. Both primer sets recovered 15 fish species in the aquarium, but MiFish‐U produced substantially more fish reads, demonstrating greater sensitivity for fish detection. This aligns with expectations as MarVer1 amplifies a broader range of vertebrates (Shaffer et al. 2025), diluting fish read counts. Since classification was restricted to Actinopterygii, potential non‐fish vertebrate reads amplified by MarVer1 were not enumerated.

Basecalling algorithms showed performance stratification in both read depth (Figure 2) and species detection (Figure 3). Fast basecalling prioritised speed but exhibited notably lower sensitivity for rare taxa; HAC balanced speed and accuracy, while SUP achieved the highest read quality (mean Q‐score 15.4) and read retention, with many samples exceeding ~100,000 raw reads per replicate (Table 3). Species detections were remarkably similar between HAC and SUP models, indicating that the extra computation time required for SUP does not necessarily translate into higher detection rates. Thus, HAC offers an optimal balance between computational efficiency and detection sensitivity for most applications.

**TABLE 3:** Comparison of basecalling models for read quality and retention.

Basecalling model	Apple M3 time	NVIDIA RTX4090 time	Mean read quality	Median read quality	Read count (millions)	% Reads > Q10	% Reads > Q15	% Reads > Q20
Fast	2 h 35 m 56 s	35 m 09 s	9.4	10.7	31.85	66.3%	0.2%	0.0%
HAC	1d 03 h 58 m 23 s	14 h 04 m 45 s	12.5	15.4	31.96	90.5%	54.2%	8.1%
SUP	16d 18 h 41 m 18 s	47 h 36 m 36 s	15.4	20.4	31.98	94.8%	83.3%	52.6%

The choice of demultiplexing tool also influenced read retention and species detection (Figure S1). In the bivariate comparison plots (Figure 3), OBITools4 and ONTbarcoder2.3 (baseline) show generally similar detection proportions across most species. However, ONTbarcoder2.3 retained more reads overall, improving the detection of rare taxa, particularly in workflows using Chelex‐extracted samples or Fast basecalling. These subtle differences in read retention may affect detections for certain species (Figure S5).

Overall, BT extraction, MiFish‐U primers and ONTbarcoder2.3 demultiplexing with HAC/SUP basecalling yield the highest detection odds across most taxa (Figures 2, 3). Chelex extraction or MarVer1 primers showed reduced performance for most species, while other factor levels exhibited milder effects. DirectPCR and QuickExtract, though not matching BT in all scenarios, still demonstrated reasonable performance when paired with MiFish‐U and either HAC or SUP, underscoring their potential as cost‐effective alternatives for many applications (Table 4).

**TABLE 4:** Workflow performance summary comparison of extraction, primers, basecalling and demultiplexing based on read yield and species recovery.

Workflow choice	Read yield	Species recovery
Extraction	BT>DirectPCR~QE>Chelex	BT>DirectPCR~QE>Chelex
Primer	MiFish‐U > MarVer1	MiFish‐U~MarVer1
Basecalling	SUP>HAC > Fast	SUP>HAC > Fast
Demultiplexing	ONTbarcoder2.3 > OBITools4	ONTbarcoder2.3 > OBITools4

### 3.2 Time, Computational Power and Other Considerations for Workflows

#### 3.2.1 Basecalling Algorithm Speed and Computation Time

Fast basecalling prioritised speed, processing a full dataset in ~30 min on an NVIDIA RTX 4090 and ~2 h on an Apple M3 Max (Table 3). HAC provided a balance between speed and accuracy, requiring ~14 h on an NVIDIA RTX 4090 and over a day on the Apple M3 Max. SUP achieved the highest read accuracy; however, this improvement came at a significant computational cost, requiring ~50 h on an RTX 4090 and over two weeks on an Apple M3 Max. The number of reads recovered and read quality scores based on the basecalling algorithm affected species detections, though the jump from HAC to SUP did not seem to be worth the nearly 4× computation time in the NVIDIA RTX 4090 or the 14× computation time on the Apple M3 Max, given only marginal gains in species detections.

#### 3.2.2 Real‐Time Species Accumulation Over 61 h

**FIGURE 4:**
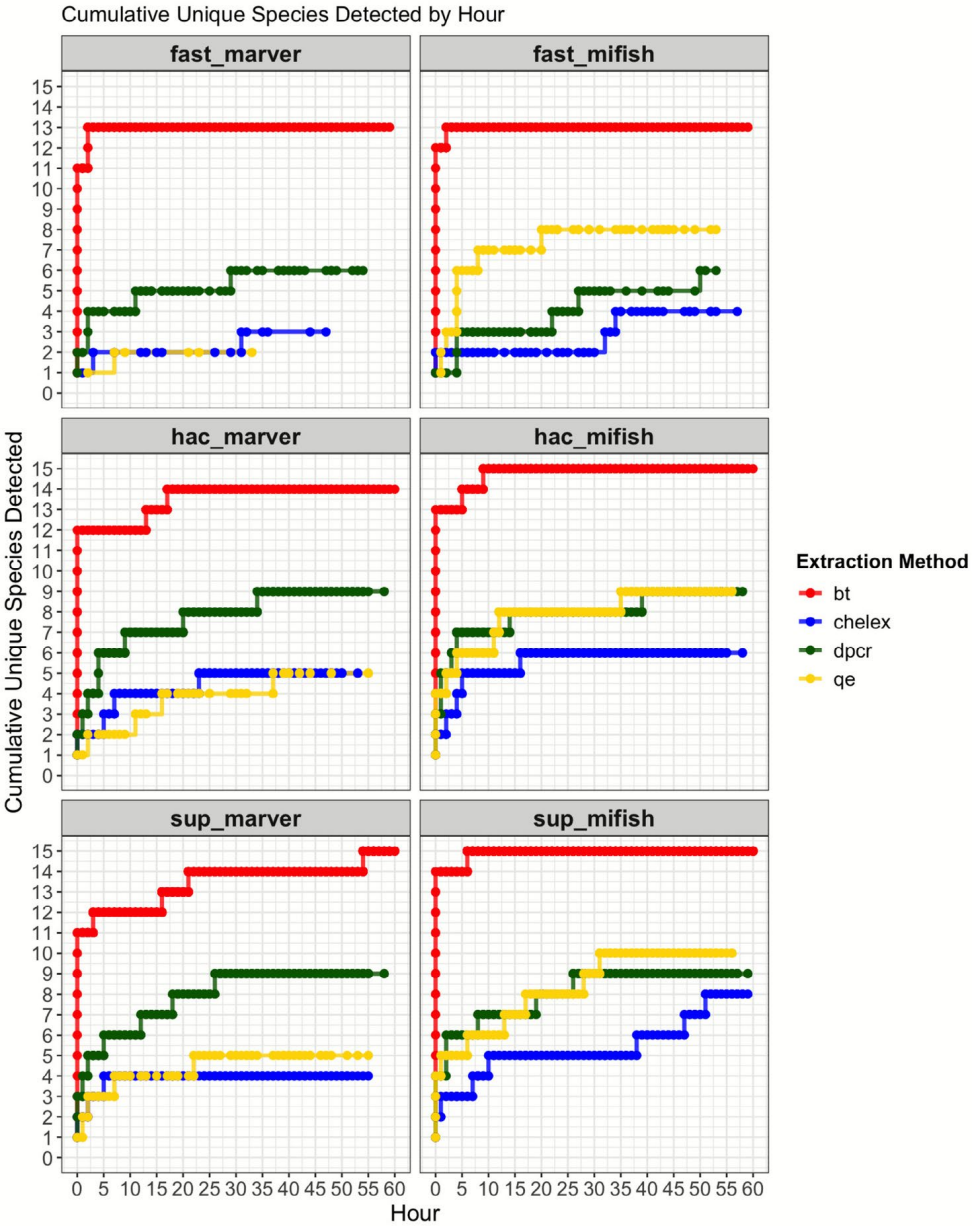
Cumulative Unique Species Detected Over Sequencing Time. The x‐axis represents sequencing time in hours, while the y‐axis indicates the cumulative number of detected species. Colour indicates DNA extraction methods: Qiagen Blood & Tissue (BT, red), Chelex (blue), DirectPCR (dPCR, green) and QuickExtract (QE, yellow), demultiplexed using OBITools4. Each facet corresponds to a combination of basecalling model (Fast, HAC, SUP) and primer set (MiFish‐U, MarVer1). PCR replicates were first pooled within each bio‐rep, followed by pooling of bio‐reps for this analysis.

**FIGURE 5:**
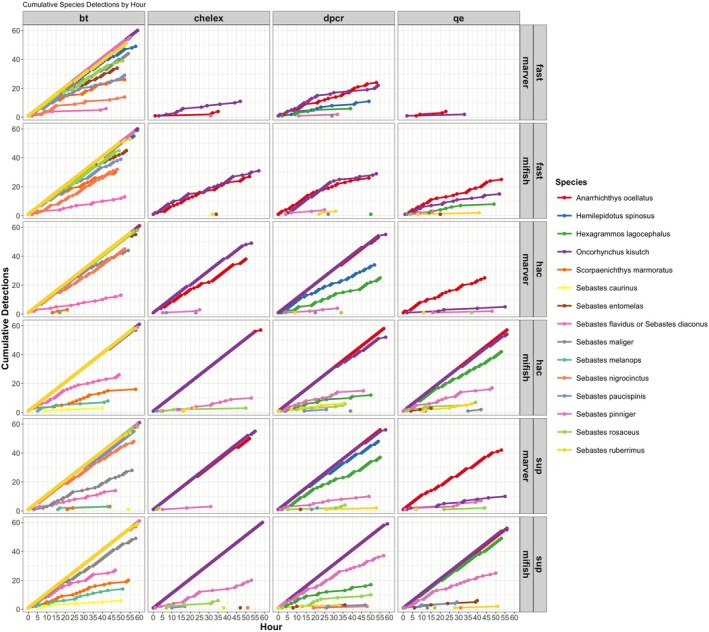
Cumulative Species Detections Over Sequencing Time. The x‐axis represents sequencing time in hours, while the y‐axis indicates the cumulative number of detections per species (colour). The 24 panels represent variations in DNA extraction method (BT, Chelex, dPCR, QuickExtract), basecalling model (Fast, HAC, SUP) and primer set (MiFish‐U, MarVer1). PCR replicates were first pooled within each bio‐rep, followed by pooling of bio‐reps for this analysis.

Cumulative species accumulation curves illustrate the rate at which the 15 unique OTUs were detected over the course of a 61‐h sequencing run (Figures 4 and 5). Sequencing duration strongly influenced detection probabilities, particularly for rare taxa such as *Sebastes ruberrimus* , which required extended sequencing times to reach stable detection thresholds. The detection rate varied across extraction methods, basecalling models and primer choices, with BT consistently outperforming all other extraction workflows in terms of rapid species recovery (Figure 4). Statistical analysis confirms that BT significantly accelerated species accumulation, with full recovery of all 15 OTUs occurring within 3–5 h across most workflows (GAM: edf = 3.908, χ^2^ = 89.16, *p* < 2e‐16). In contrast, Chelex never reached the full 15‐OTU set within 61 h; the best Chelex configuration (SUP + MiFish‐U) reached ≤ 9 OTUs by the end of the run, with other combinations recovering fewer, underscoring its inefficiency in DNA recovery (Figures 4 and 5). This delayed accumulation likely reflects lingering effects of PCR inhibitors that are not fully removed during cleanup, limiting downstream amplification despite post‐PCR purification.

BT + MiFish‐U workflows achieved rapid detections, with ≥ 12 OTUs observed by ~3–5 h for both HAC and SUP. The 15‐OTU plateau occurred at ~8–10 h with HAC and ~12–15 h with SUP (Figure 4). Even under less efficient workflow conditions (Fast basecalling or MarVer1 primers), BT samples consistently outperformed other extraction methods, with ≥ 9 OTUs detected within the first hour (Figure 4). The DirectPCR and QuickExtract extractions exhibited intermediate performance (near‐complete OTU detection within 10–12 h), while Chelex‐extracted samples detected only 6–7 OTUs by the 24‐h mark and complete detection (> 10 species) requiring over 50 h. This trend is confirmed by ZOID posterior coefficients, which were consistently negative for Chelex across multiple species (Figure 3; Figure S4).

A more granular breakdown of cumulative species detections over sequencing time is demonstrated in Figure 5, with each line representing one of the 15 detected species. The accumulation patterns reveal clear differences between species detected early versus those that appeared later. Common species, such as *Oncorhynchus kisutch* (Coho salmon), *Sebastes caurinus* (Copper rockfish) and *Hexagrammos lagocephalus* (Rock Greenling), exhibited a near‐linear accumulation curve across sequencing time, reflecting a 1:1 detection per hour rate in most workflows. These species were consistently detected early, often within the first 1–2 h of sequencing, regardless of extraction method.

In contrast, several species, including *Sebastes ruberrimus* (Yelloweye rockfish), *Sebastes melanops* (Black rockfish) and *Scorpaenichthys marmoratus* (Cabezon), showed delayed and irregular detection patterns, with steep stepwise increments in their accumulation curves. These species often required > 10 h of sequencing to achieve stable detection, particularly in DirectPCR, QuickExtract and Chelex workflows. The necessity for prolonged sequencing in these cases is attributed to their lower initial eDNA concentrations, requiring additional sequencing depth that mostly re‐reads the same finite amplicon library, rather than uncovering new molecules. While these trends suggest differences in eDNA abundance or amplification efficiency, we refer to taxa as ‘less common’ solely based on their later or less consistent detection in the sequencing run, rather than confirmed template concentrations.

Interestingly, extended sequencing times (> 40 h) did not always contribute to increased species recovery in less efficient workflows (e.g., Chelex + Fast basecalling), as species detections began to plateau despite continued sequencing. This trend suggests that rare taxa may become depleted over time, a phenomenon observed in stochastic eDNA capture processes where fewer molecules remain available for sequencing as sampling progresses. This aligns with ONT's single‐molecule sequencing mechanism, where rare molecules—once sequenced—may become depleted, particularly in workflows with limited input DNA (Hook and Timp 2023). The GAM model (edf = 3.908, χ^2^ = 89.16, *p* < 2e‐16) supports this interpretation, demonstrating a significant nonlinear relationship between sequencing time and species accumulation, with diminishing returns observed beyond 30–40 h.

Device‐level output from the MinKNOW run report shows a gradual decline in read production rate across the run, approaching a plateau in the final ~5–10 h (~55–60 h) (Figure S8, Supporting Information S5). In contrast, species‐accumulation curves for the highest‐yield workflows plateau much earlier (~3–5 h; ~10–12 h for mid‐tier), indicating that extended runtimes confer limited additional biological return.

These results underscore the critical role of DNA extraction, sequencing depth and basecalling models in optimising eDNA workflows. The greater reliability of Qiagen BT workflows, demonstrated by their consistent and rapid species recovery, may offer added value in low‐resource or high‐throughput settings. In such contexts, minimising time spent on troubleshooting and repeat runs is often more important than reducing per‐sample costs. The data suggest that short‐duration sequencing (3–6 h) is sufficient for full species recovery when using BT extraction and MiFish‐U primers, making it a robust choice for rapid eDNA applications. However, studies targeting rarer species may benefit from extended sequencing (10–12 h), particularly in workflows using DirectPCR, QuickExtract, or MarVer1 primers. Beyond 40 h, sequencing returns diminish significantly, with only marginal gains in species detections. This plateau effect is most pronounced in Chelex‐extracted samples, where even prolonged sequencing fails to recover the full species set efficiently.

## 4 Discussion

Across workflows, the combination of BT extraction, MiFish‐U primers, HAC/SUP basecalling and ONTbarcoder2.3 demultiplexing consistently recovered all 15 OTUs and reached full detection within 3–5 h. DirectPCR and QuickExtract offered field‐friendly alternatives that achieved near‐complete detection in ~10–12 h, whereas Chelex required substantially longer sequencing and recovered fewer reads. These findings contextualise the trade‐offs we discuss below.

### 4.1 Performance and Feasibility of Field‐Adaptable Extraction Methods

Our findings reinforce the pivotal role of DNA extraction methods in eDNA workflows, influencing both species recovery and practical feasibility. Across all tested conditions, Qiagen BT outperformed other methods, consistently detecting up to 15 species in the controlled aquarium setting. We also demonstrated that BT showed the strongest positive posterior coefficients across species, confirming its reliability in maximising species detection. This aligns with prior studies demonstrating that spin‐column kits maximise DNA recovery across diverse conditions due to their silica‐membrane purification, which efficiently binds DNA while removing PCR inhibitors (Kranzfelder et al. 2016; Majaneva et al. 2018). BT columns can be operated on a vacuum manifold to reduce centrifugation; however, the need for a vacuum source and power reduces portability relative to Chelex/QuickExtract (heat‐block only). As technician time is also part of cost, workflows that minimise hands‐on steps, like DirectPCR's single spin and the low‐touch heat‐block steps for QuickExtract, can be advantageous even when reagent prices are similar. DirectPCR eliminates multi‐buffer handling but still requires a high‐speed microcentrifuge. Despite the Amicon filter cost ($6.00), its total per‐sample cost is comparable in magnitude to BT once ethanol is included to QuickExtract ($4.70). We therefore treat BT as a laboratory baseline and Chelex/QuickExtract/DirectPCR as field‐adaptable options with distinct trade‐offs (Table 5).

**TABLE 5:** Time‐to‐detection across eDNA extraction methods.

Extraction Method	Hours to detect 15 species	Hours to detect 12 species	Hours to detect 9 species
BT (Qiagen)	3–5 h	1.5–3 h	< 1 h
DirectPCR	10–12 h	5–7 h	3 h
QuickExtract	10–12 h	5–7 h	3 h
Chelex	> 50 h	~24 h	~12 h

Our experimental design reflects single‐molecule sequencing paradigms rather than traditional Illumina normalisation approaches (Jain et al. 2016; Goodwin et al. 2016). The differential read yields observed across extraction methods represent genuine integrated workflow performance: a practical outcome researchers encounter when implementing these methods in field settings. Unlike Illumina sequencing, which requires equimolar pooling for cluster generation (Bentley et al. 2008), ONT directly sequences individual molecules, making throughput differences a meaningful measure of extraction efficiency rather than a technical artefact requiring correction.

Equimolar pooling is the conventional approach for Illumina component benchmarking, but it is not required for single‐molecule sequencing on the ONT platform. Our study intentionally measured integrated throughput under field‐realistic conditions, where higher‐yield extractions contribute more molecules to sequencing. Importantly, rarefaction and presence–absence sensitivity analyses yielded the same extraction rankings, demonstrating that our conclusions are robust to pooling strategy. As in most eDNA metabarcoding studies, limited post‐classification curation (e.g., collapsing indistinguishable markers or retaining genus‐level calls) is unavoidable and should be reported transparently alongside database provenance or version.

In contrast, DirectPCR and QuickExtract, while capable of recovering ~80% of species, lack targeted inhibitor removal, which may lead to reduced efficiency in high‐turbidity or inhibitor‐rich environments (Majaneva et al. 2018). Future refinements, such as additional inhibitor removal steps or pre‐concentration techniques (e.g., QuickConc; Kuroita et al. 2025), could improve DNA yield in challenging conditions. QuickConc, for instance, uses benzalkonium chloride with dispersed glass fibres to concentrate DNA and has demonstrated promising yield improvements in early trials. Similarly, employing pre‐filters or specialised extraction aids could enhance the usability of ultra‐portable methods like QuickExtract, particularly in environments with high particulate loads (Majaneva et al. 2018; Lee‐Rodriguez et al. 2024). Their ability to achieve comparable detection without the need for either cold‐chain logistics or high‐speed centrifugation makes them promising for field applications. However, DirectPCR still requires a small centrifuge, posing a barrier in ultra‐remote settings lacking stable power or benchtop equipment (Ip et al. 2024; Kirtane and Deiner 2024).

Among low‐cost alternatives, Chelex was the least reliable in our controlled aquarium system, frequently missing rarer taxa. Our study did not test inhibitor‐rich conditions; thus, this underperformance likely reflects Chelex's lower DNA retention rather than inhibition per se. Prior studies report that Chelex workflows can carry over PCR inhibitors and reduce amplification efficiency in inhibitor‐rich matrices (Walsh et al. 1991; Karlsson et al. 2022; Bracken et al. 2019), so performance may be further compromised in such settings. Chelex remains extremely inexpensive (~$0.05 per sample) which can be attractive for high‐throughput applications, even though extraction kits such as BT and QuickExtract typically cost several‐fold more. However, the trade‐off between cost and sensitivity suggests that Chelex may be better suited to scenarios where only the detection of dominant taxa is sufficient.

Ultimately, no single extraction method is universally superior, as the choice depends on study objectives and resource constraints (Table 6). Further developments in passive filtration or power‐free filtration systems (Bessey et al. 2021; Kuroita et al. 2025) could enhance eDNA workflow simplification in resource‐limited settings.

**TABLE 6:** Decision matrix for eDNA workflow optimisation based on cost, performance and suitability.

Factor	Cost	Time to detection	Sensitivity (Rare Taxa)	Infrastructure needs	Recommended for
**Extraction**					
Qiagen BT	High	3–5 h for 15 spp. (BT + HAC)	Very high	Cold storage + specialised equipment	Labs needing robust detection; can afford higher cost
DirectPCR	Medium	~10–12 h for 15 spp	Moderate	Mini‐centrifuge needed	Field contexts with modest capacity
QuickExtract (QE)	Medium	~10–12 h for 15 spp	Moderate	Minimal eqpt + moderate cold storage	Field contexts; not extreme inhibitor conditions
Chelex	Very low	> 24 h for 15 spp	Low	Minimal eqpt, no cold storage	Large‐scale screening of dominant taxa only
**Basecalling**					
Fast	Low HPC	Rapid (~35 min for a run)	Misses rare species	Standard laptop GPU helpful	Quick screening or high‐turnover monitoring
HAC	Medium HPC	~14 h for a run	Near‐SUP detection	GPU recommended	Balancing speed & accuracy for large eDNA surveys
SUP	High HPC	~2 days for a run	Highest accuracy	HPC or advanced GPU setup	SNP‐level or specialised detection needs
**Demultiplexing**					
OBITools4	Automated	—	Loses ~5%–10% reads	Command‐line, easier scripting	Large‐scale throughput (indel‐aware & quick)
ONTbarcoder2.3	Manual GUI	—	Retains more reads	No command‐line/batch possible	Rare & endangered species detection (max retention)
**Primers**					
MiFish‐U	Medium cost	Faster detection for fish	Highest fish detection	Standard eDNA lab	Fish‐only or fish‐dominated communities
MarVer1	Similar cost	Slower or~same detection	Broader vertebrate coverage	Standard eDNA lab	Multitaxa analysis, if non‐fish vertebrates matter

### 4.2 Trade‐Offs in Primer Selection: Specificity Versus Taxonomic Coverage

While DNA extraction determines the quantity and quality of eDNA recovered, primer selection further refines taxonomic resolution by influencing the breadth and specificity of species detection. As expected, the fish‐specific MiFish‐U primer set consistently outperformed MarVer1 in terms of read count, recovering approximately three times more fish reads per run. This aligns with previous studies demonstrating that primers explicitly designed for fish detection maximise on‐target amplification and improve sequencing efficiency (Plewnia et al. 2025; Tibone et al. 2025).

MarVer1, despite yielding fewer total fish reads, detected the same fish species and additionally recovered non‐fish vertebrates, which may provide a more comprehensive view of community composition. In contrast, MiFish‐U remains the optimal primer for high‐confidence fish detection with minimal off‐target amplification. In other words, the choice of primer depends on study objectives: for dedicated fish surveys, MiFish‐U is preferred, whereas for ecosystem‐wide studies where broader vertebrate diversity is important, MarVer1 can offer added value. Future research should explore whether combining multiple markers, such as 12S with mitochondrial D‐loop regions (Andruszkiewicz et al. 2020; Suarez‐Bregua et al. 2022), can improve taxonomic resolution while preserving broad ecological coverage. Moreover, advancing long‐read sequencing technologies may enable hybrid marker strategies that bridge the gap between specificity and inclusivity in species‐level assignments. (Chang et al. 2024; Domingo‐Bretón et al. 2024).

Since both markers target short 12S mini‐barcodes, a few congeners remained unresolved. Resolution can be improved by adding a longer or complementary locus (e.g., COI ~650 bp or 12S + D‐loop). However, this usually trades off amplification success and extends runtime in field‐portable workflows because eDNA copy number declines with fragment length in seawater (Brandão‐Dias et al. 2025; West and Deagle 2025). Although Nanopore enables long reads, practical recovery of long eDNA fragments in field‐portable settings remains challenging; recent PCR‐free mito reconstructions from eDNA required higher DNA input and more computational resources than we target here (Mizuno et al. 2025). A pragmatic path is hybrid designs that retain a short 12S locus for robust detection and add a longer locus only where template length permits. Database scope is a key portability consideration. For example, MitoFish enables laptop‐class analyses for fishes, whereas comprehensive vertebrate databases increase storage and runtime by orders of magnitude; teams expanding beyond fishes should budget for those costs or precompute databases.

### 4.3 Computational Trade‐Offs: Basecalling Accuracy and Demultiplexing Efficiency

Basecalling and demultiplexing are critical steps in eDNA workflows, each introducing trade‐offs between speed, accuracy and species recovery. Our results highlight how basecalling models impact detection efficiency, with Fast mode significantly reducing processing time (< 1 h) but at the cost of lower quality scores. These lower quality reads often lack the resolution needed for confident species‐level identification, leading to higher uncertainty in taxonomic assignments and effectively dropped from downstream analyses, contributing to inconsistent detection of low‐abundance taxa. HAC basecalling, in contrast, maintains a strong balance, recovering nearly all target species while completing basecalling within a reasonable timeframe (~14 h). Recent studies have demonstrated that HAC‐optimised basecalling with R10.4.1 flow cells improve sequence accuracy by reducing homopolymer errors, which previously limited Nanopore species identification (Ni et al. 2023). Although SUP provides the highest read accuracy and retention, its extended processing time (two days to two weeks) makes it impractical for most field or real‐time applications.

These findings align with previous research demonstrating that modern ONT basecallers, particularly when paired with R10.4.1 flow cells and updated chemistries, can approach Illumina‐level accuracy (Chang et al. 2024; Stoeck et al. 2024; Dierickx et al. 2024). In rapid‐response contexts, such as invasive species detection, Fast basecalling may be sufficient when only major taxa are of interest. However, for comprehensive biodiversity surveys targeting rare or cryptic species, HAC remains the preferred option, offering an optimal balance between speed and sensitivity. SUP may be necessary for specialised applications such as population genetics, where the highest basecalling fidelity is required.

Beyond basecalling, demultiplexing approaches further shape species recovery patterns, influencing whether low‐abundance taxa are retained or lost. OBITools4, which trims primers, tags and barcodes in a single command, provides a highly automated workflow suited for large‐scale studies. While OBITools4 is indel aware and offers greater automation, this efficiency comes with trade‐offs: consistently retaining fewer reads than ONTbarcoder2.3, potentially leading to missed detections of rare taxa. The observed difference in read retention (~5%–10% more for ONTbarcoder2.3) could directly impact whether a species is detected, particularly in eDNA samples with extremely low DNA concentrations.

For large‐scale monitoring programmes or studies requiring rapid processing of high‐throughput sequencing data, OBITools4 remains a valuable tool for streamlining analysis. However, in scenarios where every read is critical, such as pathogen surveillance or the detection of endangered species, ONTbarcoder2.3's more manual but higher‐recovery approach may be preferable. Future refinements, such as adjusting OBITools4's mismatch thresholds or integrating read‐recovery scripts, could help bridge the gap between automation and sensitivity, ensuring that computational trade‐offs do not compromise biodiversity assessments. Although Dorado includes integrated demultiplexing, we did not assess it here; a direct, controlled evaluation against ONT amplicon tag sets is warranted as the feature stabilises.

### 4.4 Detection Plateau in Real‐Time eDNA Sequencing

Our hourly analysis over a 61‐h nanopore sequencing run revealed how quickly detection of the 15 known aquarium OTUs (representing the known aquarium species) plateaued under different workflows. In optimal scenarios (BT + MiFish‐U + HAC), nearly all species were consistently detected within 3–5 h. These short timeframes are particularly valuable for real‐time ecological monitoring, as field teams could stop sequencing once a detection plateau is reached, conserving resources without compromising species recovery.

Conversely, suboptimal workflows showed a much slower species detection, often requiring ≥ 24 h for detection to stabilise and > 50 h to recover only a partial species set. This highlights a direct link between method efficiency and sequencing duration, where lower DNA yields or increased sequencing errors can prolong the time needed to achieve full species detection. Extending sequencing time does not compensate for poor extraction efficiency. If DNA yields are too low or degraded at the start, additional sequencing contributes minimal new detections and only increases computational costs.

Our findings suggest that implementing adaptive stopping during sequencing protocols can optimise run times. For routine biodiversity surveys, sequencing 3–5 h is sufficient using high‐yield workflows (BT + MiFish‐U + HAC). For low‐abundance species, sequencing ~10–12 h may improve detection. Beyond 40 h, further sequencing is unlikely to be cost‐effective, as additional species detections plateau at < 5% new recoveries. This plateau likely reflects a combination of (i) a limited number of unique amplicons after PCR, (ii) random resampling when taxa are very rare and (iii) gradual loss of active pores. However, it is important to note that our study focused on detecting 15 target OTUs (species‐equivalent units). In broader biodiversity surveys, such as community‐wide freshwater or marine assessments, extended sequencing might still yield new detections beyond 40 h, particularly for rare or low‐abundance taxa. These insights reinforce the idea that real‐time sequencing analytics can optimise run duration dynamically, allowing researchers to adaptively terminate sequencing once species accumulation stabilises, thereby reducing costs and computational burden (Chang, Ip, Ng, and Huang 2020; Plewnia et al. 2025).

### 4.5 Towards Accessible and Scalable eDNA Monitoring in Resource‐Limited Settings

Given these findings, we outline key considerations for scaling eDNA workflows to resource‐limited settings, focusing on logistics and methodological accessibility. Our data show that simplified extractions (DirectPCR, QuickExtract) and high accuracy basecalling (HAC) can still recover most known taxa, making them viable alternatives when working under budget or infrastructure constraints. Notably, BT workflows may also offer added value in low‐resource settings by minimising troubleshooting and reruns, which can outweigh per‐sample cost savings. These results align with previous studies demonstrating that Nanopore‐based metabarcoding can approximate Illumina's performance at lower cost and with reduced laboratory requirements (Tibone et al. 2025; Veillat et al. 2024; Stoeck et al. 2024; Kirchgeorg et al. 2024).

From a capacity‐building perspective, integrating cost‐effective extractions (DirectPCR, QuickExtract) with portable MinION sequencers in a ‘lab‐in‐a‐box’ format (Watsa et al. 2020; Chang, Ip, Ng, and Huang 2020) could significantly expand global eDNA adoption, particularly in biodiversity monitoring programmes in the Global South, where infrastructure and technical expertise may be limited (Hirsch et al. 2024). In addition to logistical benefits, localised sequencing enables greater data sovereignty, allowing researchers to control their own workflows and analyses without relying on centralised sequencing facilities. Recent hardware, such as ONT MinION Mk1D, allows direct sequencing from an iPad. In this configuration, Fast basecalling is the practical real‐time choice. HAC/SUP basecalling generally requires a high‐spec laptop or desktop GPU and is best run post hoc. Users who need real‐time decisions should plan around Fast basecalling mode.

Despite these advances, several challenges remain in making eDNA workflows fully field deployable. Achieving cold‐chain independence, standardising protocols for inhibitor‐rich samples, and ensuring adequate training on simplified extraction methods are ongoing barriers (Rieder et al. 2024). While ONT sequencing can be performed offline using preconfigured settings, internet access is still required for licence verification, software updates, and troubleshooting, limiting the feasibility of fully independent field deployments. Expanding support for offline configurations would further enhance the portability of eDNA sequencing (Geckeler et al. 2025). Addressing these constraints through clear standard operating procedures (SOPs), remote training programmes, and improved sample preservation techniques will be critical for broader implementation (Hirsch et al. 2024; Rieder et al. 2024; Stammnitz et al. 2024).

Beyond sequencing, bioinformatics accessibility remains a major hurdle for eDNA‐users without coding training. Many analysis pipelines rely on command‐line tools, limiting adoption in non‐specialised labs. However, cloud‐based platforms, graphical user interface (GUI)‐driven tools and low‐cost field‐deployable computational resources are emerging as viable alternatives (Bloomfield et al. 2024; Ip et al. 2023). Recent advancements in affordable, low‐power GPUs and edge computing devices now enable real‐time basecalling and analysis on a budget. Bloomfield et al. (2024) demonstrated that Nanopore sequencing could be implemented without on‐site bioinformaticians using a GPU costing only $649, highlighting the feasibility of decentralised, cost‐effective sequencing analysis. Such approaches, when combined with cloud‐based Galaxy‐tools bioinformatics platforms (Abueg et al. 2024) and workflow automation software like APSCALE (Buchner et al. 2022), significantly lower financial barriers, computational expertise and hardware barriers for non‐experts. Similarly, CrocoBLAST (Tristão Ramos et al. 2017) provides an online GUI classifier, enabling species identification without the need for command‐line Kraken2, BLASTn, or other taxonomic assignment tools. These advancements are instrumental in bridging the bioinformatics accessibility gap, making low‐cost, scalable eDNA analysis more inclusive. Our study directly evaluates these simplified approaches, providing empirical support for their viability in resource‐limited field settings.

Emerging low‐power, field‐adaptable molecular tools could further enhance eDNA accessibility. Isothermal amplification methods (e.g., RPA) offer an attractive alternative to traditional PCR, reducing power demands and simplifying workflow requirements (Plewnia et al. 2025). When integrated with in situ sequencing, such innovations could enable near real‐time eDNA assessments, expanding the feasibility of biodiversity monitoring in remote environments. While such approaches were beyond the scope of this study, they represent logical next steps in the continued democratisation of eDNA methodologies, ensuring that species monitoring remains scalable, cost‐effective and accessible, even in the most infrastructure‐limited environments.

## 5 Conclusion

Across 48 workflow combinations in a controlled aquarium, BT + MiFish‐U with HAC/SUP approached full detection most rapidly (≥ 12 species by ~3–5 h; plateau ~8–15 h).

DirectPCR and QuickExtract reached near‐complete detection in ~10–12 h with minimal infrastructure, whereas Chelex was less sensitive and requires > 24 h to reach close‐to‐similar detection levels. HAC balances speed and accuracy; MiFish‐U increases fish‐specific read depth compared, with MarVer1's broader vertebrate scope.ONTbarcoder2.3 retained more low abundance reads than OBITools4, which traded a small sensitivity loss for greater automation. Real‐time sequence accumulation curves support adaptive stopping once plateaus are reached.

Overall, these results illustrate that no single workflow is universally optimal, but rather that each method offers distinct advantages that can be matched to specific study goals. These findings provide a transparent decision framework for researchers to navigate trade‐offs between detection sensitivity, cost efficiency and sequencing speed based on study constraints. By identifying where meaningful losses occur, we highlight the conditions under which cost‐effective or rapid workflows can maintain reliable species detection and where they risk compromising accuracy. Rather than simply making eDNA workflows cheaper or faster, our goal was to clarify what is gained or lost with each methodological choice, ensuring that researchers can optimise their workflows based on project priorities. By clearly mapping the trade‐offs between cost, time and detection accuracy, this study empowers researchers to make informed methodological choices, ensuring that eDNA monitoring remains scalable, efficient, and adaptable across diverse ecological and logistical settings.

## Author Contributions

Conceptualisation: Y.C.A.I., E.A.A., R.P.K.; Methodology: Y.C.A.I., E.A.A.; Formal analysis: Y.C.A.I., with inputs from E.A.A., R.P.K.; Writing – original draft: Y.C.A.I.; Writing – review and editing: E.A.A., R.P.K., S.L.H.; Funding acquisition: E.A.A., R.P.K.

## Funding

This work was supported by OceanKind (GR042390), David and Lucile Packard Foundation (GR016745).

## Disclosure

Benefits Sharing: This research was designed with the explicit goal of democratising molecular monitoring by developing streamlined, low‐cost eDNA workflows suitable for remote or resource‐limited settings. All protocols, datasets, and benchmarking results are made openly available via public repositories to support capacity building across diverse global research communities. The study does not involve access to genetic resources governed under the Nagoya Protocol, but the methods are intentionally generalisable and intended for broad international use. By reducing logistical and financial barriers to eDNA‐based monitoring, this work contributes to non‐monetary benefit‐sharing by enabling environmental research and conservation applications in under‐resourced regions. The open‐access nature of the tools, as well as accompanying outreach and documentation, serves as key components of benefit‐sharing under the Nagoya framework.

## Conflicts of Interest

The authors declare no conflicts of interest.

## Supporting information


**Data S1:** men70091‐sup‐0001‐Supinfo.docx.

## Data Availability

Basecalled Fast, HAC and SUP FASTQ files have been deposited in the NCBI Sequence Read Archive under BioProject accession PRJNA1311144. All other data supporting the findings of this study (including sample metadata, demultiplexing outputs and summary tables) are provided in the main text and its Supporting Information. In accordance with confidentiality requirements, we do not archive UMI tag sequences or tag‐design code; tags were designed based on unpublished guidance from Eric Coissac (personal communication).
