# Supplementary material for: Fast, Flexible, Feasible: A Transparent Framework for Evaluating eDNA Workflow Trade‐Offs in Resource‐Limited Settings
Source: Mol Ecol Resour. 2026 Jan 3;26(1):e70091. doi: 10.1111/1755-0998.70091 (PMC12760245; doi:10.1111/1755-0998.70091)
Supplement: Supplementary file 1 — Data S1: men70091‐sup‐0001‐Supinfo.docx. [file MEN-26-e70091-s001.docx]

9. Supporting Information

Supplementary Material 1. Compilation of all extraction protocols

Supplementary Material 2. Parameters and codes for ONTbarcoder2.3.

Supplementary Material 3. Parameters and codes for OBITools4.

Supplementary Material 4. Full list of ZOID posterior coefficients, bio_rep independent observations, aggregated pcr_rep

Supplementary Material 5. Full MinKNOW run report output

Supplementary Table S1. Aquarium species roster (as provided by aquarium staff) and mapping of Kraken2 12S assignments to the analysis OTUs used in this study. ‘Status’ indicates whether the 12S call matched the rostered taxon or was curated to the appropriate OTU when 12S cannot distinguish congeners or when database aliases/coverage warranted remapping. Two congeneric pairs were treated as single OTUs (*Hemilepidotus* *hemilepidotus* + *H.* *spinosus*; *Sebastes* *flavidus* + *S*. *diaconus*). The ‘Reasons’ column documents the basis for each curation (e.g., 12S indistinguishability, missing 12S record in MitoFish, or out-of-range database alias).

| Aq SN | Visual Observations (Aquarium Species) | eDNA-12S Detections | Status | Reasons |
| --- | --- | --- | --- | --- |
| 1 | *Anarrhichthys ocellatus* | *Anarrhichthys ocellatus* | Match | Verified detection. Direct species match. |
| 2 | *Hemilepidotus hemilepidotus* | *Hemilepidotus hemilepidotus* | Match | Verified detection. Direct species match. |
| 3 | *Hemilepidotus spinosus* | *Hemilepidotus hemilepidotus (combined)* | Correction | 12S resolution at genus level; species-level misclassification expected (Miya et al., 2015). Besides, this is not represented in MitoFish database. |
| 4 | *Hexagrammos lagocephalus* | *Hexagrammos decagrammus* | Correction | 12S marker cannot fully resolve *Hexagrammos* species (Miya et al., 2015; Kai et al., 2014). Both species co-occur in similar habitats. |
| 5 | *Oncorhynchus kisutch* | *Oncorhynchus kisutch* | Match | Verified detection. Direct species match. All other *Oncorhynchus* congenerics were collapsed into *Oncorhynchus* *kisutch*. |
| 6 | *Rhinogobiops nicholsii* | *-* | Missed | Species not detected in eDNA. Possibly due to low shedding rates or sequencing bias (Miya et al., 2015). |
| 7 | *Scorpaenichthys marmoratus* | *Scorpaenichthys marmoratus* | Match | Verified detection. Direct species match. |
| 8 | *Sebastes caurinus* | *Sebastes cheni* | Correction | Both species belong to the Pteropodus rockfish group. Likely a misclassification due to high mitochondrial sequence conservation (Hyde & Vetter, 2007). |
| 9 | *Sebastes diaconus* | *Sebastes fasciatus* | Correction | *S*. *fasciatus* is an Atlantic species, unlikely in this tank. More likely detection corresponds to Pacific S. diaconus (Love et al., 2002). Besides, this is not represented in MitoFish database. |
| 10 | *Sebastes flavidus* | *Sebastes fasciatus* (combined) | Correction | Same correction as above: *S*. *fasciatus* is an Atlantic species, while Pacific *S*. *flavidus* or *S*. *diaconus* are more plausible. |
| 11 | *Sebastes entomelas* | *Sebastes iracundus* | Correction | Both species belong to the Sebastosoma rockfish group and have similar 12S mitochondrial sequences (Rocha-Olivares et al., 1999). |
| 12 | *Sebastes maliger* | *Sebastes mentella* | Correction | *S*. *mentella* is an Atlantic species, misidentified due to sequence similarity. The correct Pacific species in the tank is *S*. *maliger* (Love et al., 2002). |
| 13 | *Sebastes melanops* | *Sebastes oblongus* | Correction | *S*. *oblongus* is an Asian species; *S*. *melanops* is a Pacific species. Likely misclassification due to conserved mitochondrial sequences (Hyde & Vetter, 2007). |
| 14 | *Sebastes nebulosus* | *-* | Missed | Species not detected in eDNA, likely due to low shedding or sequencing depth limitations (Miya et al., 2015). |
| 15 | *Sebastes nigrocinctus* | *Sebastes schlegelii* | Correction | *S*. *schlegelii* is an Asian species; *S*. *nigrocinctus* is a Pacific species. Likely a misclassification due to mitochondrial similarity (Rocha-Olivares et al., 1999). |
| 16 | *Sebastes paucispinis* | *Sebastes paucispinis* | Match | Verified detection. Direct species match. |
| 17 | *Sebastes pinniger* | *Sebastes macdonaldi* | Correction | *S*. *macdonaldi* is a deep-water species. The correct match based on habitat and geography is *S*. *pinniger* (Love et al., 2002). |
| 18 | *Sebastes rosaceus* | *Sebastes steindachneri* | Correction | Likely misidentification due to mitochondrial conservation between these *Sebastes* species (Rocha-Olivares et al., 1999). |
| 19 | *Sebastes ruberrimus* | *Sebastes scythropus* | Correction | *S.* *scythropus* is a non-Pacific species. Correct match is *S*. *ruberrimus* (Love et al., 2002). |


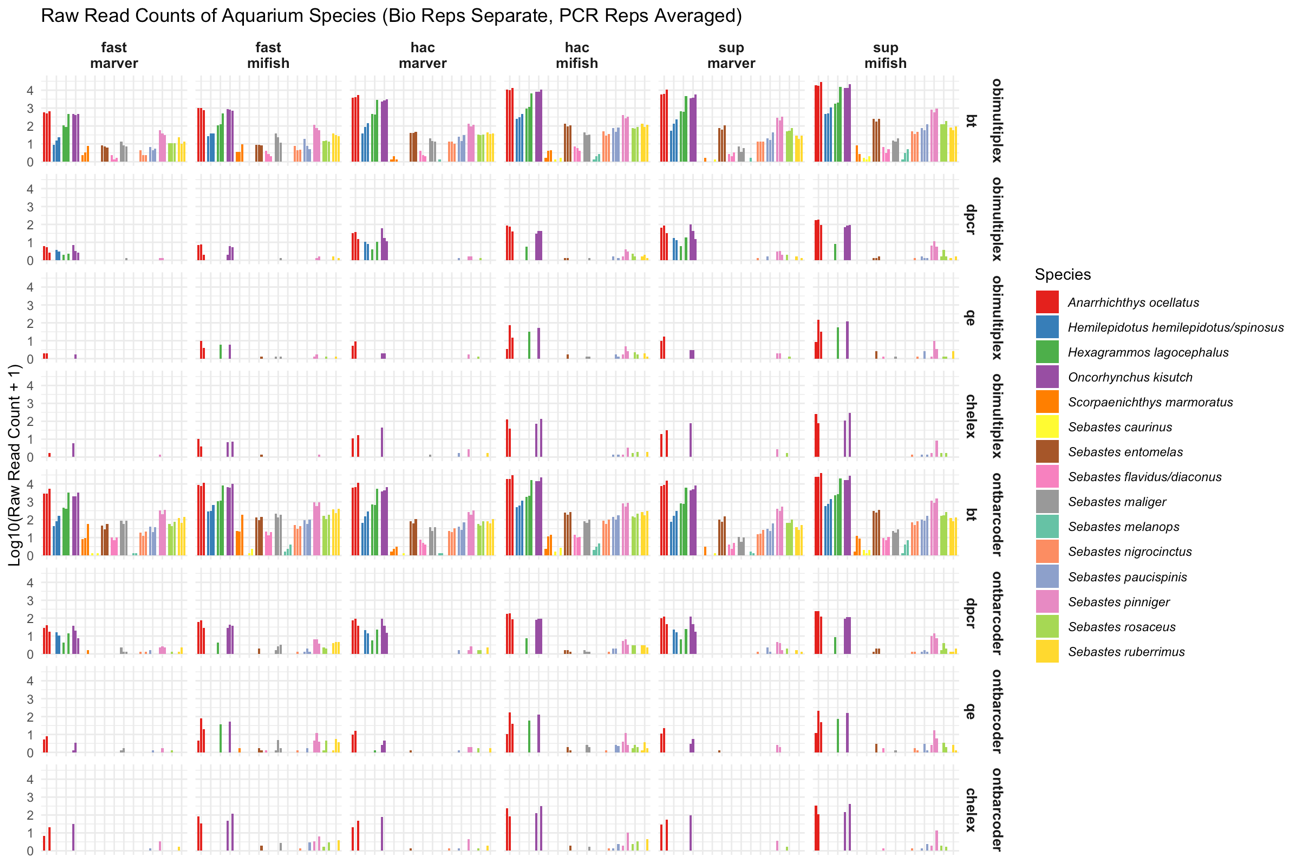


Supplementary Figure S1. Read Counts Across Workflow Variables in eDNA Analysis (Biological Replicates Separate, PCR Replicates Averaged). In contrast to Figure 2 in the main text, which shows only ONTbarcoder2.3 outputs, this supplementary figure includes both OBITools4 and ONTbarcoder2.3 results and averages PCR replicates within each biological replicate. Bar plots displaying the log10-transformed raw read counts of detected aquarium species across different workflow components, including demultiplexing methods (OBITools4, ONTbarcoder2.3), basecalling models (FAST, HAC, SUP), primers (MiFish-U, MarVer1), and DNA extraction methods (Qiagen Blood & Tissue (BT), Chelex, QuickExtract (QE), and DirectPCR (dPCR)). Biological replicates (bt1, bt2, bt3) are presented separately here, while PCR replicates (a, b, c) were averaged within each biological replicate. Each species appears as up to three side-by-side bars (or fewer if not detected in all replicates).


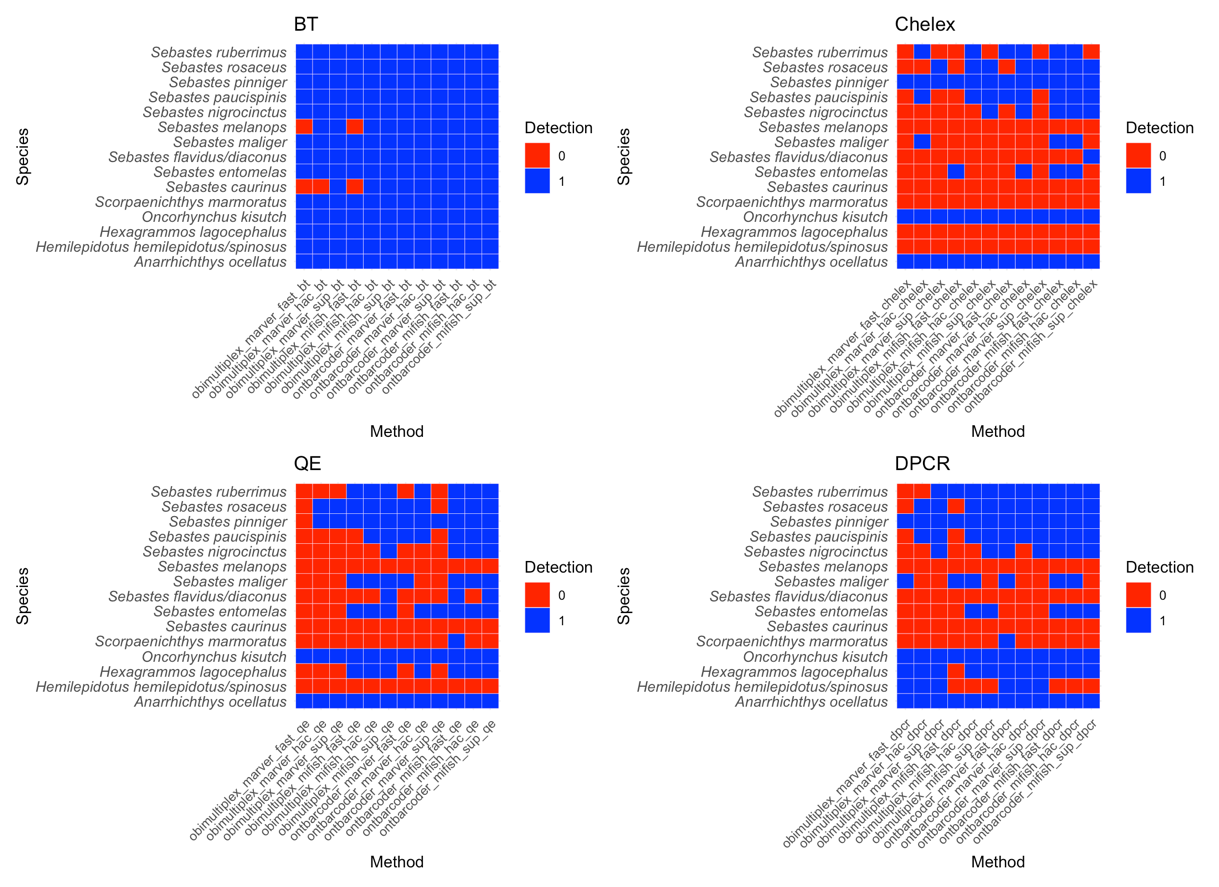

Supplementary Figure S2. Binary Detection Heatmap of Aquarium Species Across eDNA Extraction Methods. This heatmap visualizes species detection (presence: blue = 1; absence: red = 0) across four DNA extraction methods: Qiagen Blood & Tissue (BT), Chelex, QuickExtract (QE), and DirectPCR (dPCR). Each panel corresponds to an extraction method, with species listed on the y-axis and methodological variations, including basecalling models, demultiplexing tools, and primers, on the x-axis.


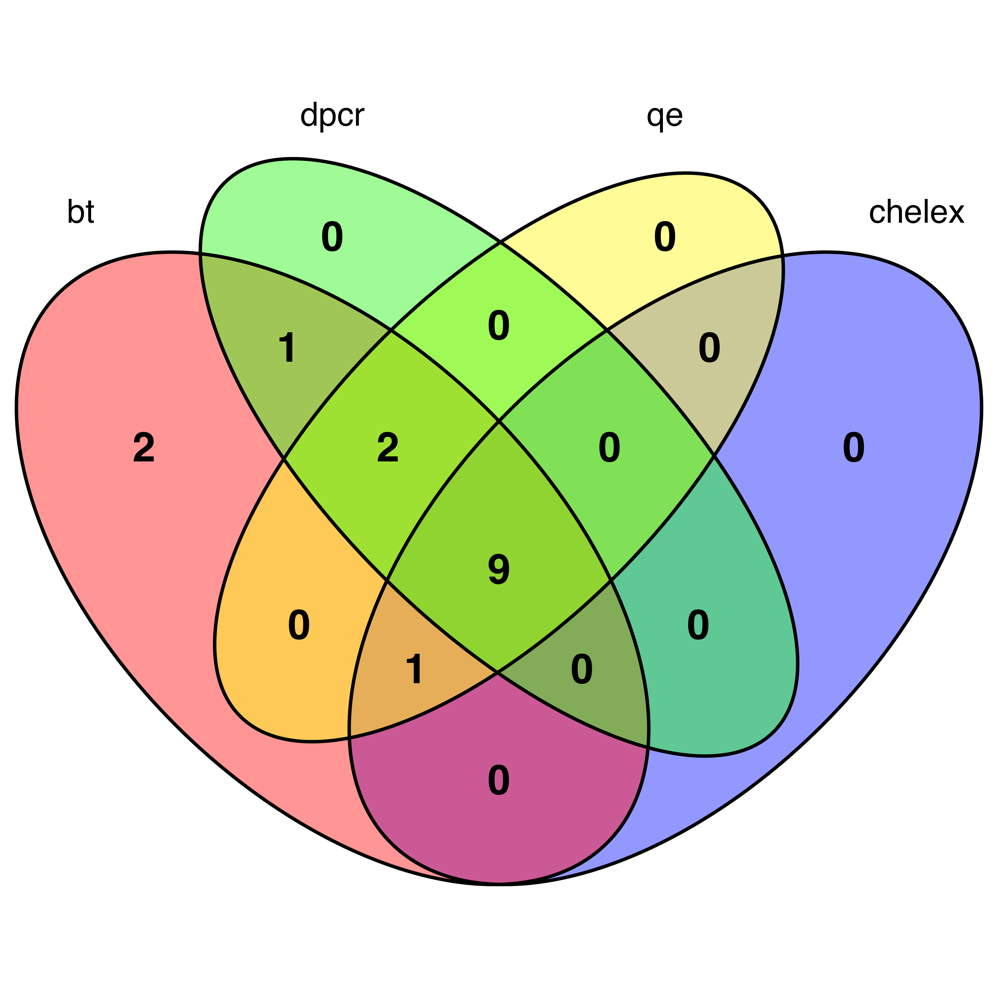

Supplementary Figure S3. Overlap of Detected Species Across eDNA Extraction Methods.

Venn diagram illustrating the number of unique and shared species detections across four DNA extraction methods: Qiagen Blood & Tissue (BT), DirectPCR (dPCR), QuickExtract (QE), and Chelex.


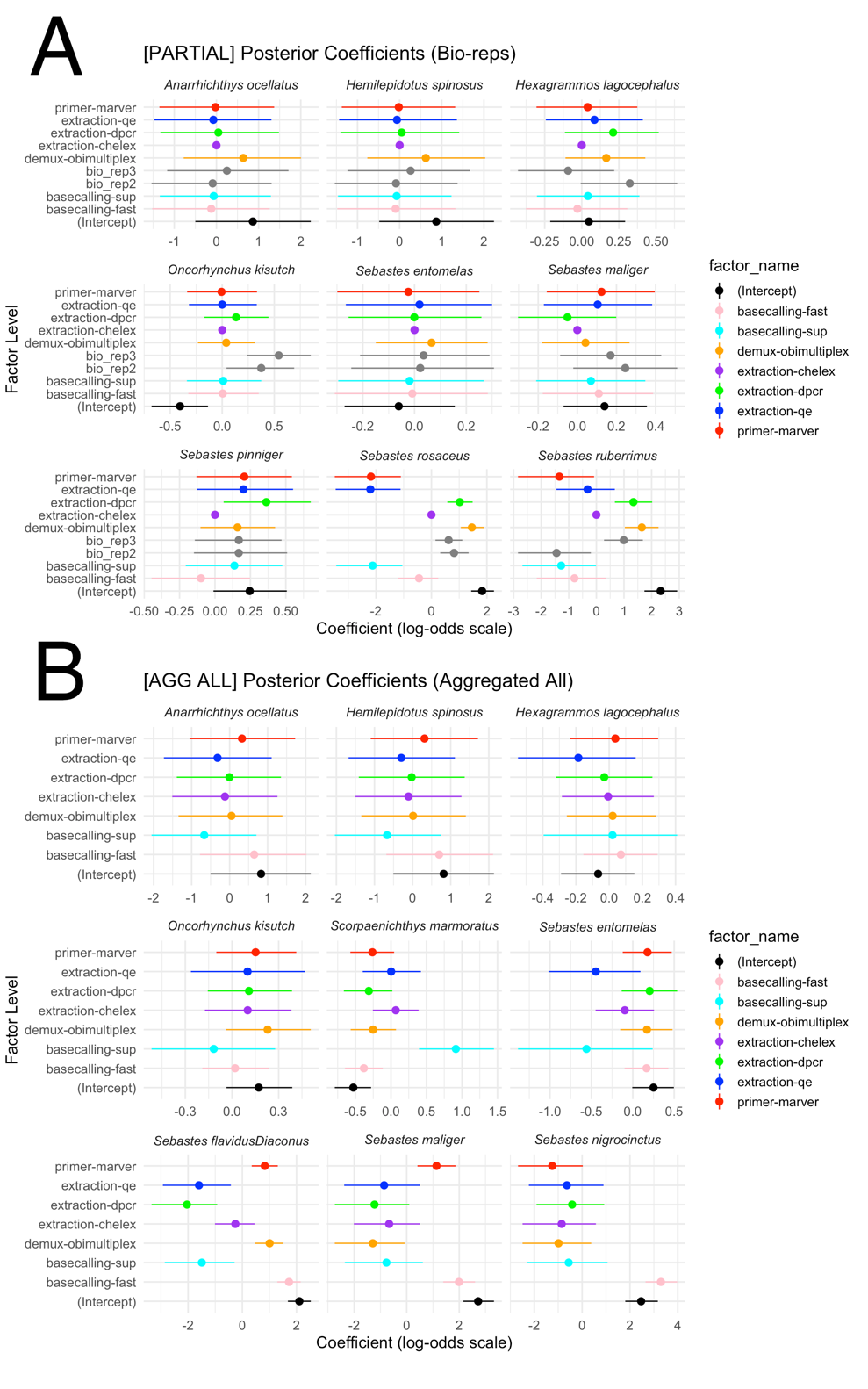


Supplementary Figure S4. Posterior ZOID coefficients for each species under the fully aggregated analysis, in which PCR and biological replicates were normalized and then averaged to produce a single data point per sample. Each panel corresponds to one of the target species, and each colored point represents the posterior mean log-odds estimate (relative to the baseline workflow of BT extraction, MiFish-U primer, HAC basecalling, and ONTbarcoder2.3 demultiplexing). Horizontal lines show the 95% credible intervals for each factor level. Positive values indicate increased detection odds compared to baseline, while negative values indicate decreased detection odds. This full aggregation approach reduces within-sample variability, revealing subtle effects that might otherwise be obscured by replicate-level noise. With the two panels are shown, Panel A displays ZOID coefficients under the fully aggregated analysis (PCR and biological replicates averaged), whereas Panel B displays coefficients from the partial-aggregation analysis where biological replicates are retained as independent observations.


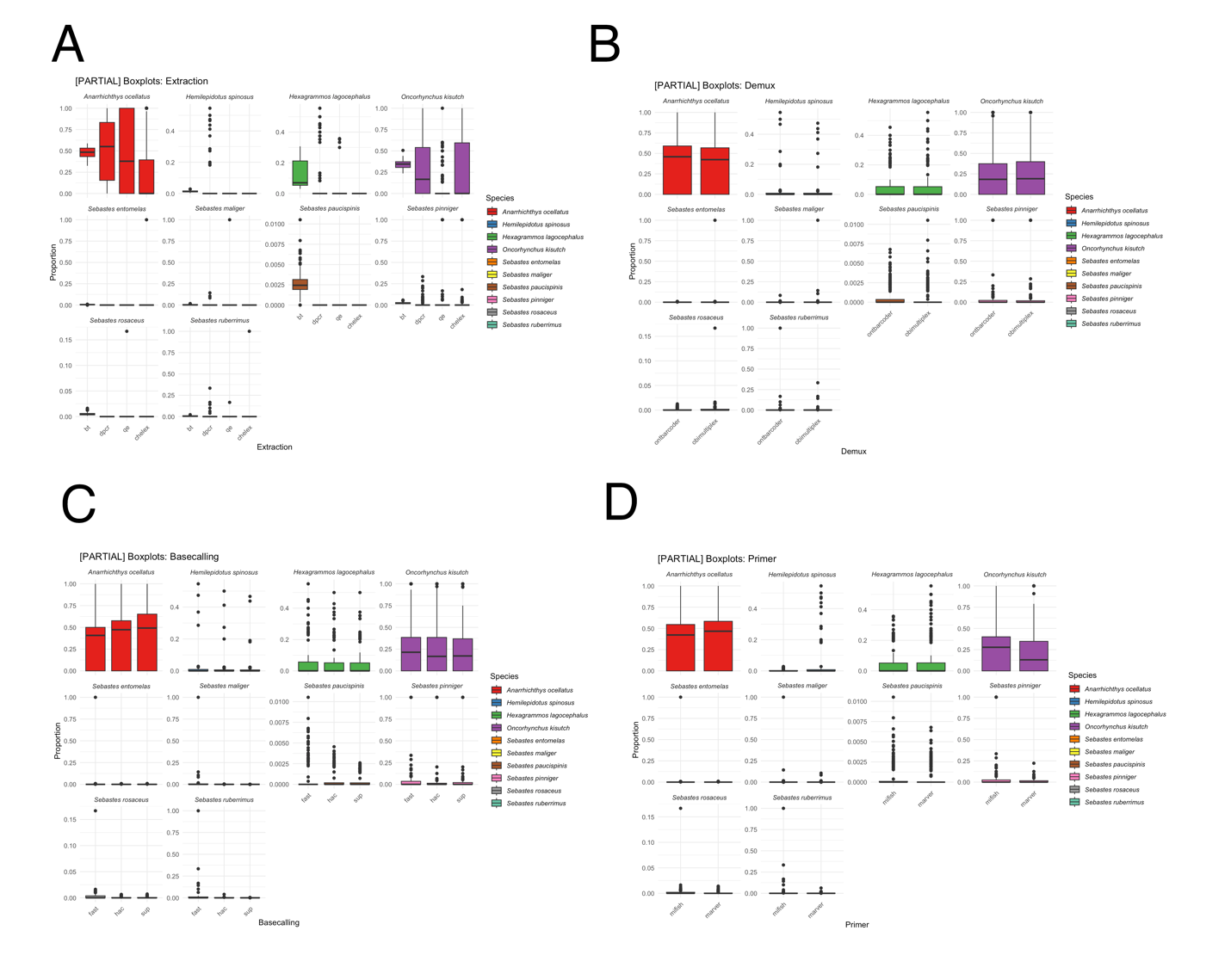


Supplementary Figure S5. Boxplots of species-level proportions across major workflow factors. Boxplots display proportional read abundance (y-axis) for 10 representative species across four workflow components: (A) DNA extraction method, (B) demultiplexing tool, (C) basecalling model, and (D) primer set. Each facet shows how individual species respond to different methodological choices, based on ZOID-modeled proportional data. Only a subset of species and factors are shown here for clarity, full results are in Supplementary Material 4.


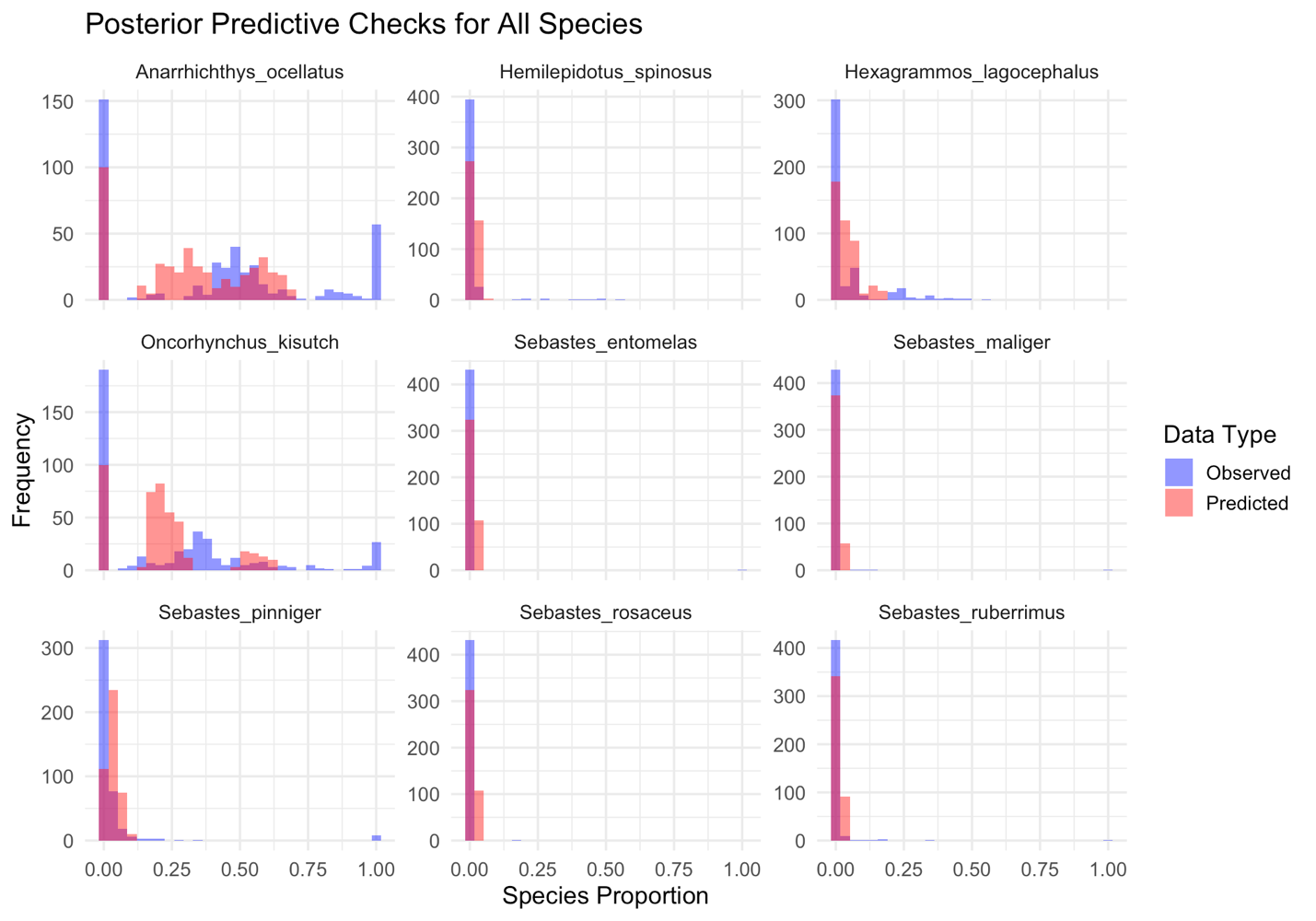


Supplementary Figure S6. Posterior predictive checks for species-level read proportions across all modeled taxa using the ZOID Bayesian hierarchical model. Histograms show the distribution of observed (blue) and model-predicted (red) species proportions across samples for each species. Bars are semi-transparent; where observed and predicted bins overlap, the colors blend (appearing purple/darker), indicating agreement. Close overlap between observed and predicted distributions indicates good model fit, while mismatches—such as in Oncorhynchus kisutch and Anarrhichthys ocellatus—suggest over- or under-dispersion not fully captured by the model. These checks help identify taxa with systematic deviations and assess the robustness of species-level inferences.

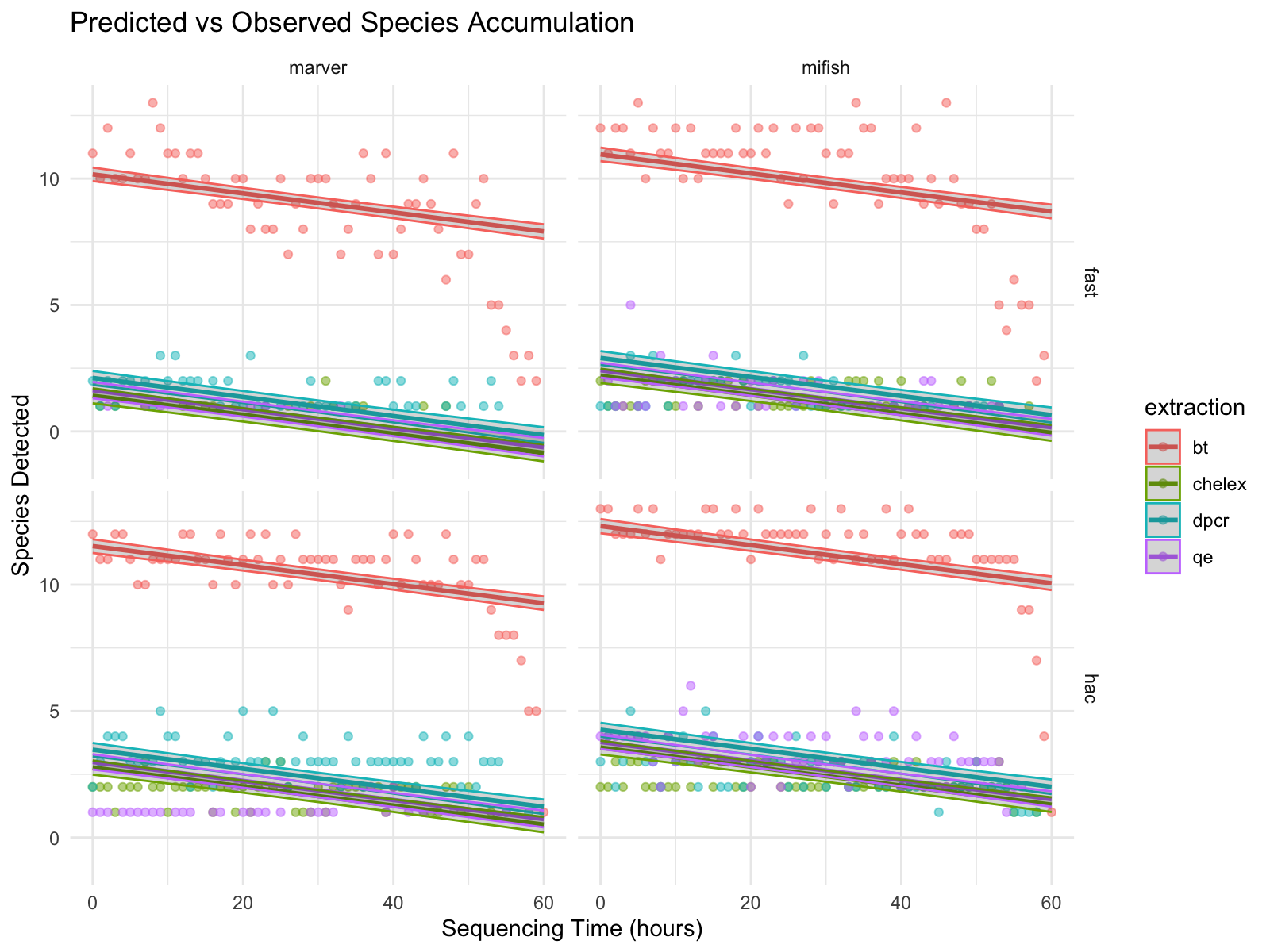

Supplementary Figure S7. Species accumulation over sequencing time for alternative extraction methods, primer sets, and basecalling modes. Each panel plots the number of fish species detected at hourly intervals (points) against sequencing time (hours), with colored lines showing the fitted generalized additive model (mgcv) for each extraction method (BT = red, Chelex = green, DirectPCR = teal, QuickExtract = purple). Columns correspond to primer sets (left: MarVer1; right: MiFish‑U) and rows to Nanopore basecalling models (top: Fast; bottom: HAC). The downward slopes illustrate how detection of new species tapers off as sequencing progresses, with high‑yield workflows (BT) maintaining higher species counts throughout the run.


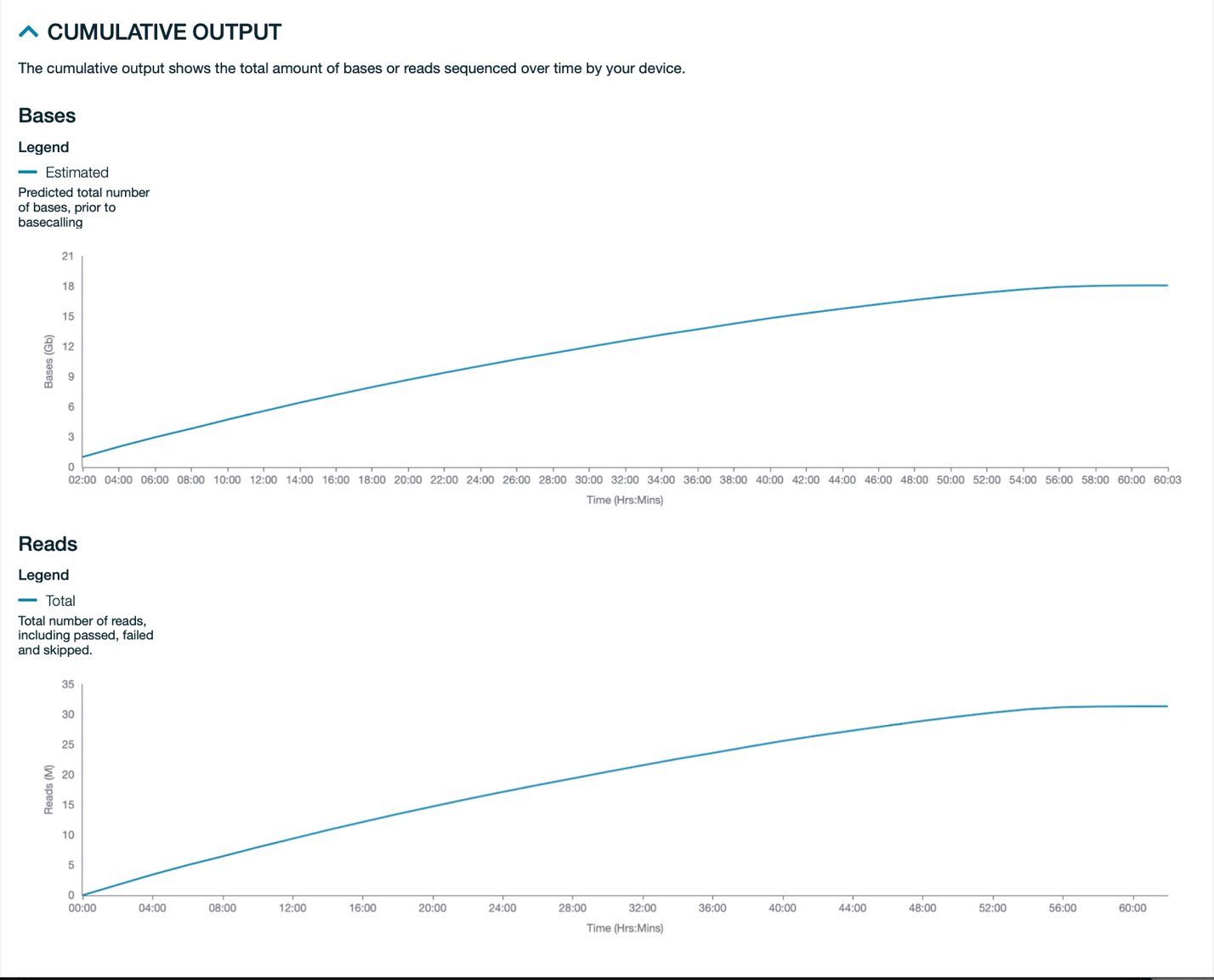


Supplementary Figure S8. Device-level cumulative output over 61-hour MinION sequencing run. Cumulative bases (Gigabases, upper panel) and reads (Millions, lower panel) produced by the MinION flow cell over time, exported from MinKNOW run report. Output shows progressive accumulation with plateauing in the final ~5–10 hours (55–60 h). Full MinKNOW run report provided in Supplementary Material 5.


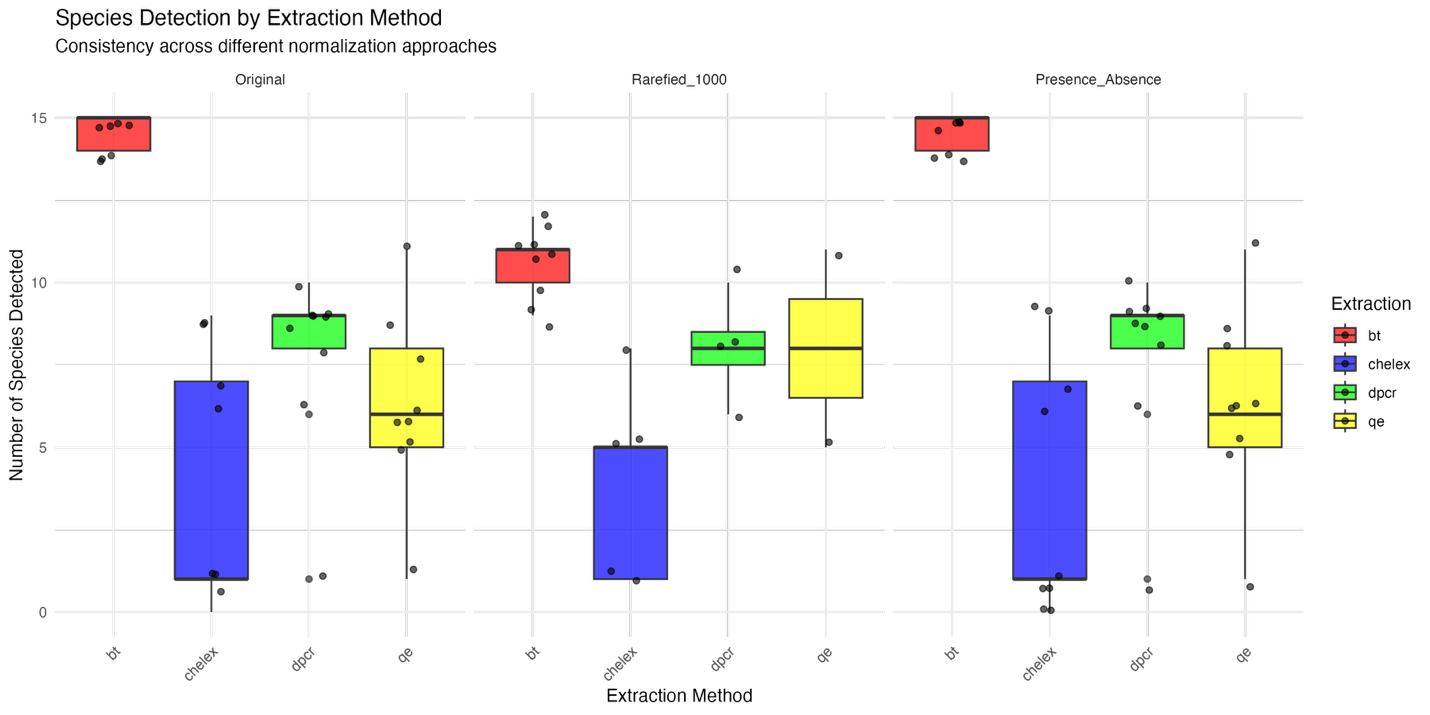

Supplementary Figure S9. Species detection by extraction method across different normalization approaches. Boxplots show number of species detected per biological replicate (n=3 per method) under three analytical approaches: original (non-normalized), rarefied to 1000 reads per sample, and presence-absence (binary detection). Extraction method rankings remain consistent across all approaches (BT > DirectPCR ≈ QuickExtract > Chelex), with statistically significant differences maintained in each analysis (Kruskal-Wallis p < 0.001). Points represent individual biological replicates; boxes show median and interquartile range.
